# Pathogenicity assessment of Shiga toxin-producing *Escherichia coli* strains isolated from wild birds in a major agricultural region in California

**DOI:** 10.3389/fmicb.2023.1214081

**Published:** 2023-09-26

**Authors:** Michelle Qiu Carter, Beatriz Quiñones, Nicole Laniohan, Diana Carychao, Antares Pham, Xiaohua He, Michael Cooley

**Affiliations:** ^1^Produce Safety and Microbiology Research Unit, U.S. Department of Agriculture, Agricultural Research Service, Western Regional Research Center, Albany, CA, United States; ^2^Foodborne Toxin Detection and Prevention Research Unit, U.S. Department of Agriculture, Agricultural Research Service, Western Regional Research Center, Albany, CA, United States

**Keywords:** comparative pathogenomics, Shiga toxins, heat-labile toxin, cytotoxicity, biofilms, virulence genes, pathogenicity islands, avian *Escherichia coli*

## Abstract

Shiga toxin-producing *Escherichia coli* (STEC) consists of diverse strains differing in genetic make-up and virulence potential. To better understand the pathogenicity potential of STEC carried by the wildlife, three STEC and one *E. coli* strains isolated from wild birds near a major agricultural region in California were selected for comparative pathogenomic analyses. Three American crow (*Corvus brachyrhynchos*) strains, RM9088, RM9513, and RM10410, belonging to phylogroup A with serotypes O109:H48, O9:H30, and O113:H4, respectively, and a red-winged blackbird (*Agelaius phoeniceus*) strain RM14516 in phylogroup D with serotype O17:H18, were examined. Shiga toxin genes were identified in RM9088 (*stx*_1a_), RM10410 (*stx*_1a_ + *stx*_2d_), and RM14516 (*stx*_2a_). Unlike STEC O157:H7 strain EDL933, none of the avian STEC strains harbored the pathogenicity islands OI-122, OI-57, and the locus of enterocyte effacement, therefore the type III secretion system biogenesis genes and related effector genes were absent in the three avian STEC genomes. Interestingly, all avian STEC strains exhibited greater (RM9088 and RM14516) or comparable (RM10410) cytotoxicity levels compared with EDL933. Comparative pathogenomic analyses revealed that RM9088 harbored numerous genes encoding toxins, toxins delivery systems, and adherence factors, including heat-labile enterotoxin, serine protease autotransporter toxin Pic, type VI secretion systems, protein adhesin Paa, fimbrial adhesin K88, and colonization factor antigen I. RM9088 also harbored a 36-Kb high pathogenicity island, which is related to iron acquisition and pathogenicity in *Yersinia* spp. Strain RM14516 carried an acid fitness island like the one in EDL933, containing a nine gene cluster involved in iron acquisition. Genes encoding extracellular serine protease EspP, subtilase cytotoxin, F1C fimbriae, and inverse autotransporter adhesin IatC were only detected in RM14516, and genes encoding serine protease autotransporter EspI and P fimbriae were only identified in RM10410. Although all curli genes were present in avian STEC strains, production of curli fimbriae was only detected for RM9088 and RM14516. Consistently, strong, moderate, and little biofilms were observed for RM9088, RM14516, and RM10410, respectively. Our study revealed novel combinations of virulence factors in two avian strains, which exhibited high level of cytotoxicity and strong biofilm formation. Comparative pathogenomics is powerful in assessing pathogenicity and health risk of STEC strains.

## 1. Introduction

Shiga toxin-producing *Escherichia coli* (STEC) consists of a group of genetically and phenotypically diverse strains differing greatly in pathogenicity. Some strains can cause severe diseases in humans, while others are only associated with mild diarrhea or no disease at all (Coombes et al., 2011; National Advisory Committee on Microbiological Criteria for Foods, 2019). Such variation in disease outcome is attributed in part to the differences in the STEC genetic makeup, especially the repertoire of virulence genes. Mutation and horizontal gene transfer (HGT) are two main forces shaping the virulence evolution in STEC. Mutations that result in subpopulations with improved fitness under a particular selective pressure are known as adaptive mutations (Rosenberg, 2001; Notley-McRobb et al., 2003), such as enhanced nutrient-scavenging, increased resistance to stresses or antibiotics, or improved pathogenicity in bacterial pathogens, a phenomenon known as patho-adaptation (Sokurenko et al., 1999; Maurelli, 2007). In *E. coli* and *Shigella* spp., adaptive mutations have been described for silencing the synthesis of lysine decarboxylase, production of type I fimbrial adhesin FimH, as well as biogenesis of curli fimbriae (Sakellaris et al., 2000; Casalino et al., 2003; Hommais et al., 2003; Ronald et al., 2008). Loss-of-function mutations in genes encoding global stress regulator RpoS, the response regulator of the two-component signal transduction system RcsB, and the auto-aggregative protein Cah are widespread in STEC populations. However, the direct impact of such mutations on the STEC pathogenicity requires further investigation (Carter et al., 2012, 2014, 2018).

Unlike mutations, HGT can accelerate bacterial adaptation considerably because bacteria acquire novel traits from non-parental sources and even from distantly related lineages (Arnold et al., 2022). *E. coli* has great ability to obtain foreign DNA via transformation, transduction, or conjugation. In fact, several key virulence factors in enterohemorrhagic *Escherichia coli* (EHEC), a subset of STEC associated with severe human illnesses including hemorrhagic colitis (HC) and hemolytic uremic syndrome (HUS), are all associated with mobile genetic elements (MGEs), including prophages for Shiga toxin expression, the locus of enterocyte effacement (LEE) pathogenicity island (PAI) for the A/E lesion formation and biogenesis of type III secretion system (T3SS), and a large virulence plasmid (pEHEC) for enterohemolysin production. Additionally, several phenotypic traits that contribute to survival and persistence of STEC in diverse ecological niches are encoded by the genes co-located on the same DNA fragments known as genomic islands (GIs). In addition to LEE and pEHEC, there are several GIs and PAIs in STEC conferring advantages in all aspects of survivability, including attachment and biofilm formation, nutrient uptake and metabolism, and stress or antibiotic resistance. For examples, the acid fitness island (AFI) provides the bacteria the ability to survive under extreme acidic conditions (Mates et al., 2007). The locus of heat resistance (LHR) confers resistance to heat, chlorine, and oxidizing agents (Mercer et al., 2017). The tellurite resistance genes in STEC are co-located with the gene encoding the Iha adhesin on a large GI known as tellurite resistance- and adherence-conferring island (TRI) (Tarr et al., 2000; Taylor et al., 2002). More recently, a large GI, the locus of adhesion and autoaggregation (LAA), was revealed in a subset of LEE negative STEC strains, and some of these strains could cause HC or HUS (Montero et al., 2017). The high pathogenicity island (HPI), first discovered in *Yersinia pestis* (Carniel et al., 1991), is involved in iron storage and uptake, and present mainly in *Yersinia* strains with high pathogenicity profiles (Carniel et al., 1996; Carniel, 1999). Homologs of HPI were reported in enteroaggregative *E. coli* (EAEC) as well as in several STEC clinical isolates (Schubert et al., 1998; Karch et al., 1999).

Although ruminant animals, primarily cattle, are main reservoirs of STEC, other domestic animals and wildlife including birds are important reservoirs or spillover hosts of STEC (Persad and LeJeune, 2014; Kim et al., 2020). Wild birds were first identified as a source of STEC in 1997 (Wallace et al., 1997), in which, 1–3% of bacterial isolates recovered from fecal samples of wild birds were STEC O157:H7. Ever since, diverse STEC strains have been detected and isolated from various bird species including pet birds worldwide (Kobayashi et al., 2002, 2009; Neher et al., 2016; Sanches et al., 2017; Navarro-Gonzalez et al., 2020; Seleem et al., 2021). Birds could disseminate STEC into environments and/or transmit STEC to other animals including humans directly or indirectly. Examining wildlife living close to cattle and pig farms in Denmark revealed that cattle and a starling (*Sturnus vulgaris*) harbored identical STEC strains, implying a role of wild birds in STEC transmission (Nielsen et al., 2004). A similar observation was reported in the United States; STEC O157:H7 recovered from starlings near several dairy farms at a distinct geographical location were genotypically indistinguishable from the STEC O157:H7 strains recovered from cattle in the dairy farms (Williams et al., 2011). In fact, transmission of STEC O157:H7 from STEC-positive birds (European starlings) to the STEC-negative birds and to cattle were demonstrated under experimental conditions (Kauffman and LeJeune, 2011). In a recent study that investigated the shedding dynamics of foodborne pathogens by wild birds on farmlands in California, identical pathogen strains including STEC O157, O26, and O103, were shared episodically among birds and between wild geese and free range cattle, suggesting the presence of a common source of contamination in pre-harvest environments and potential transmission between species (Navarro-Gonzalez et al., 2020).

Shiga toxin-producing *Escherichia coli* is one of the most common bacterial pathogens linked to the fresh produce-associated outbreaks (Herman et al., 2015; Bennett et al., 2018; Kintz et al., 2019). In the United States, several nationwide outbreaks associated with leafy greens have been traced to pre-harvest contamination. Potential sources of contamination for STEC include soil, water, manure, wild and domestic mammals, birds, rodents, and insects (Gutierrez-Rodriguez and Adhikari, 2018). Following the large 2006 spinach associated outbreak of STEC O157:H7 infection (Sharapov et al., 2016), isolates from feral swine, cattle, surface water, sediment, and soil near a spinach field in California were matched to the outbreak strain and surface water was determined to be the most likely route of contamination (Cooley et al., 2007; Jay et al., 2007). More recently, canal water in the Yuma farming region in Arizona was identified as a potential source of contamination for the large 2018 romaine lettuce associated outbreak of STEC O157:H7 infection (Bottichio et al., 2020), while agricultural water and cattle adjacent to a lettuce field in the California Central Coast growing region were suspected to be the sources of contamination for the two multi-state romaine lettuce associated outbreaks of STEC O157:H7 infection during 2018–2019 (Waltenburg et al., 2021).

Information about the prevalence and pathogenicity potential of STEC in pre-harvest environments, including wildlife, is needed for the development of effective mitigation strategies. In a 2-year survey study that investigated the prevalence of common enteric pathogens in the vicinity of California Central Coast growing region, 11% of the samples (*n* > 1,000) tested positive for STEC (Cooley et al., 2014). A similar study that examined the prevalence of common foodborne pathogens in vegetable farms in New York State revealed that 2.7% of the samples tested positive for STEC (*n* > 500) (Strawn et al., 2013). Although diverse serotypes and genotypes of STEC strains have been isolated from different bird species, knowledge about the pathogenesis of STEC other than serotypes O157:H7, O104:H4, and the “Big Six” non-O157 serogroups (O26, O45, O103, O111, O121, and O145) is limited. In this study, we selected three STEC and one *E. coli* strains recovered from wild birds in the Salinas Valley, an important leafy greens-growing region in California, United States, to assess the pathogenicity potential of these avian strains by comparative pathogenomics and phenotypic assays. Our data revealed the presence of novel combinations of virulence determinants in the avian STEC strains, such as the hybrid pathotype of STEC-ETEC (enterotoxigenic *E. coli*). The high level of cytotoxicity and strong biofilm formation in two avian STEC strains implies the emergence of STEC strains that are potentially capable of causing severe human illnesses in the pre-harvest environments.

## 2. Materials and methods

### 2.1. Bacterial strains and growth media

*E. coli* strains and their sources are listed in Table 1. Strains RM9088, RM9513, RM10410, and RM14516 were isolated by cloacal swab from captured birds (*Corvus brachyrhynchos* and *Agelaius phoeniceus*) in a major agricultural region in California as described previously (Cooley et al., 2013). Wildlife sampling at all locations was approved under a set of California Department of Fish and Game (CDFG) Scientific Collection Permits issued to USDA Wildlife Services and CDFG personnel contracted to collect the samples and ship to USDA in Albany, California. Additionally, a federal permit with the U.S. Fish and Wildlife Services was obtained for sampling of geese, crows, and blackbirds. The wildlife sampling was conducted through a contract with state and federal wildlife agencies using their standard protocols. The strains were grown routinely in Luria-Bertani (LB) broth (10 g tryptone, 5 g yeast extract, and 5 g NaCl per liter) (BD Difco).

**TABLE 1:** Genomic characteristics of *E. coli* strains used in this study.

Strains	^a^Sources (Location, year)	^b^Serotype	Phylogroup/Genotype	*stx* genes	Chromosome (bp)/ GenBank Accession #	^c^Plasmids (bp)/GenBank Accession #		References
						pEHEC	Others	
EDL933	Ground beef (USA, 1982)	O157:H7	E/ST11	*stx*_1a_ + *stx*_2a_	5,528,445/AE005174.2	92,077/AF074613.1	N/A	Burland et al., 1998; Perna et al., 2001
MG1655	Stool (USA, 1922)	O16:H48	A/ST10	N/A	4,641,652/U00096.3	N/A	N/A	Blattner et al., 1997
RM9088	Crow (USA, 2009)	O109:H48	A/ST339	*stx*_1a_	5,270,611/CP042298.1	167,256/CP042296.1	86,529/CP042297.1	Carter et al., 2019b
RM9513	Crow (USA, 2009)	O9:H30	A/ST1294	N/A	4,614,872/CP042352	N/A	93,878/CP042351	This study
RM10410	Crow (USA, 2009)	O113:H4	A/ST10	*stx*_1a_ + *stx*_2d_	5,227,472/CP042350	N/A	N/A	Carter et al., 2019b
RM14516	Blackbird (USA, 2009)	O17:H18	D/ST6507	*stx*_2a_	4,957,901/CP043937	107,620/CP043938	N/A	This study

^a^The source and isolation time for strain MG1655 is based on the information available for its parental strain K-12 as described previously.

^b^The serotype of strain MG1655 is determined by *in silico* typing using the O antigen and H antigen database described previously.

^c^pEHEC refers to the plasmid carrying genes encoding enterohemolysin.

### 2.2. Genome sequencing, annotation, and analyses

Bacterial DNA was extracted from the mid-exponential phase cultures grown in LB broth as described previously (Miller et al., 2005). Briefly, cells were lysed with SDS followed by sequential treatment with RNase A and proteinase K. The DNA was first precipitated in a sodium acetate/ethanol solution, and then purified by phenol/chloroform extraction, followed by the final ethanol precipitation. The purified DNA was re-suspended in Qiagen Elution Buffer (QIAGEN) for genome sequencing. Genomic libraries were prepared according to the PacBio (Pacific Biosciences) 20 kb library standard protocol using SMRT-bell DNA template prep kit 3.0, followed by size-selection with BluePippin size selection systems (Sage Science, Inc.), and then with P6v2 template binding step. DNA sequencing was performed on an RS II instrument (Pacific Biosciences, Menlo Park, CA, United States) with P6/C4 sequencing chemistry, and 360-min data collection protocol. The sequence reads were filtered with PreAssembler Filter prior to *de-novo* assembly with RS_HGAP_Assembly v.3. The complete genome sequences were submitted to GenBank for annotation using Prokaryotic Genome Annotation Pipelines. The GenBank accession numbers are listed in Table 1. The phylogroups were determined using the Clermont method (Clermont et al., 2013). Multi-locus sequence typing (MLST) was conducted using MLST 2.0 service at the Center for Genomic Epidemiology with the *Escherichia coli* #1 configuration (Achtman 7 gene scheme, *adk, fumC*, *gyrB*, *icd*, *mdh*, *purA*, and *recA*) (Wirth et al., 2006). The serotypes were determined by BLASTn searches of *E. coli* O-antigen and H-antigen gene databases described previously (Wang et al., 2003; Iguchi et al., 2015).

### 2.3. Identification of virulence genes

*E. coli* virulence genes listed in The Virulence Factor DataBase (VFDB) as of December 2022 were used as queries of BLASTn to search a custom database containing all genomes examined in this study. The listed genes are present in *E. coli* pathotypes belonging to adherent invasive *E. coli* (AIEC), avian pathogenic *E. coli* (APEC), EAEC, Shiga toxin-producing enteroaggregative *E. coli* (StxEAEC), EHEC, enteropathogenic *E. coli* (EPEC), ETEC, neonatal meningitis-associated *E. coli* (NMEC), and uropathogenic *E. coli* (UPEC) (Supplementary Table 1). The BLASTn was performed in Geneious Prime^®^ software (Dotmatics) with a threshold of 65% for gene coverage and 70 or 25% for sequence identity at nucleotides or amino acids level, respectively.

### 2.4. Detection of GIs and PAIs

Three GIs and seven PAIs known to contribute to stress resistance and/or pathogenicity in enteric pathogens as described previously (Carter et al., 2022a,b) were examined by performing BLASTn searches against a custom database containing all genomes described in this study. The three GIs included AFI, LHI, and TRI; the seven PAIs included LEE, OI-57, OI-122, LAA, HPI, locus of proteolysis activity (LPA), and subtilase encoding pathogenicity island (SE-PAI). When a complete GI or PAI was not detected, each coding sequence (CDS) encoded by the query GI or PAI was used to search the genome of the testing strain by BLASTP to reveal if any homologs were present in the genome of the testing strain. Presence of key virulence genes on each PAI were revealed by BLASTn of each virulence gene followed by mapping their chromosomal locations with the Geneious Prime^®^ software. Strain EDL933 harbors two TRIs, OI-43 and OI-48, which exhibit over 99% identity. The OI-43 was used as the query in this study.

### 2.5. Comparative analysis of Stx-prophages

The complete genome sequence of each avian STEC strain was submitted to PHASTER (Arndt et al., 2016) for identification of prophage and prophage-like elements. The putative integration sites were initially identified by PHASTER and confirmed in Geneious Prime^®^ software using “Find Repeats” in the defined chromosomal regions. The Stx-prophage genomes were extracted from the corresponding bacterial genomes. Comparative analysis was performed in Geneious Prime^®^ software. Briefly, Stx-prophages genomes were aligned using Geneious Alignment with the following parameters: Alignment type: Global alignment with free end gaps; Cost Matrix: 65% similarity; Gap open penalty: 12; Gap extension penalty: 3; and Refinement iterations: 2. A neighbor-joining consensus tree was constructed with the following parameters: Genetic Distance Model, Jukes-Cantor; Resampling Method, bootstrap; and number of replicates, 10,000.

### 2.6. Curli production and biofilm formation

Curli fimbriae were examined by growing each strain at 26°C for 1 or 2 days on the Congo Red indicator (CRI) plates, consisting of LB agar plates without sodium chloride and supplemented with 40 μg/ml of Congo Red dye and 10 μg/ml of Coomassie Brilliant Blue, as described previously (Carter et al., 2011). Curli-producing strains were indicated by red colonies whereas curli-deficient strains were indicated by white colonies on CRI plates. Biofilm assays were carried out as described previously (Carter et al., 2016, 2018). Briefly, one ml of LB broth inoculated with 1 × 10^6^ cells/ml was aliquoted into a borosilicate glass tube and then incubated statically at 28°C for 24, 48, and 120 h. At the end of each incubation, the planktonic cells were removed carefully, and the tubes were rinsed twice with one ml sterile distilled water and then stained with one ml 0.1% crystal violet at room temperature for 30 min. The dye was then removed gently, and the tubes were washed twice with sterile distilled water. The crystal violet bound to the glass tube was solubilized in 0.5 ml of 33% acetic acid and the absorbance was determined at 570 nm using a microplate reader (SpectraMax 340; Molecular Devices, Sunnyvale, CA). Tubes with uninoculated media served as negative controls. Each data set was the average of results from at least three biological replicates. All data were first evaluated for normal distribution by the Shapiro-Wilk test using Graph Pad Prism 10 software (Dotmatics). The difference in biofilm formation, represented by the absorbance at 570 nm, among the avian strains was assessed by the adjusted *P*-value of the Tukey’s multiple comparisons test after a One-way ANOVA test (*P ≤ 0.05; **P ≤ 0.01; ***P ≤ 0.001; ****P ≤ 0.0001). Similarly, the difference in biofilm formation of each strain at various incubation times was assessed by the adjusted *P*-value of the Tukey’s multiple comparisons test after a One-way ANOVA test.

### 2.7. Cytotoxicity assay

The Stx activity of the avian *E. coli* strains was measured using a mammalian Vero cell line, Vero-d2EGFP, that harbored a destabilized variant (*t*_1/2_ = 2 hr) of the enhanced green fluorescent protein (EGFP) as described previously (Quiñones et al., 2009; Quiñones and Swimley, 2011). Strains EDL933 and K-12 sub-strain MG1655 were used as a positive and a negative control for the assay, respectively. Cell-free culture supernatants for all tested strains were prepared as previously described (Quiñones et al., 2009; Quiñones and Swimley, 2011; Silva et al., 2019). One-day prior to intoxication, Vero-d2EGFP cells were seeded at 9,500 cells per well in Greiner Bio-One CELLSTAR^®^ black 96-well microplates with μClear^®^ bottoms (VWR International) and were grown at 37°C under humidified conditions with 5% CO_2_ in Ham’s F-12 Nutrient medium (Life Technologies Corp.), supplemented with 10% fetal bovine serum (American Type Culture Collection) and 1% penicillin-streptomycin (Life Technologies). The Vero-d2EGFP cells were then exposed to Ham’s F-12 medium containing tenfold dilutions of cell-free culture supernatants from each bacterial strain and were incubated at 37°C for 20 h in a 5% CO_2_ humidified incubator. Following intoxication, the Vero-d2EGFP cells were briefly rinsed three times with 1X phosphate-buffered saline, and the EGFP fluorescence from the Vero-d2EGFP cells was measured using a BioTek Synergy HT Multi-Detection Microplate Reader (Agilent Technologies) with the 485/20 nm excitation filter and the 528/20 nm emission filter. All experiments were performed in triplicate and each experimental condition consisted of six replicates. The results were expressed as percentages of the GFP fluorescence values for culture supernatant-treated Vero-d2EGFP cells when compared to the fluorescence values from control Vero-d2EGFP cells incubated without bacterial supernatants. The significant differences in the cytotoxicity levels among the STEC strains were determined with a One-way ANOVA test followed by a Tukey-HSD *post hoc* analysis (*P*-value < 0.05) in R version 4.2 (R Core Team, 2022).

## 3. Results

### 3.1. Genomic characteristics of avian *E. coli* strains

Unlike STEC O157:H7 strain EDL933, *in silico* phylo-typing placed the three *E. coli* crow strains, RM9088, RM9513, and RM10410, in phylogroup A, while the blackbird *E. coli* strain RM14516 in phylogroup D (Table 1). For the genomes of the three crow strains, different plasmid profiles were detected. RM9088 contained two large plasmids and one of them harbored enterohemolysin genes (*hlyCABD*), thus was designated as pEHEC; RM9513 contained one large plasmid that lacked the enterohemolysin genes; while no plasmids were identified in RM10410. These three crow strains also carried different *stx* genes and had different serotypes and genome types, determined by the MLST-based sequence types (STs) (Table 1). Strain RM9088 carried the *stx*_1a_ genes only, had a serotype of O109:H48, and belonged to ST339; strain RM9513 did not carry any *stx* genes, had a serotype of O9:H30, and belonged to ST1294; and strain RM10410 carried both *stx*_1a_ and *stx*_2d_ genes, had a serotype of O113:H4, and belonged to ST10, the same sequence type as the *E. coli* K-12 sub-strain MG1655. Like strain EDL933, the genome of the blackbird strain RM14516 was composed of a chromosome and a pEHEC. This blackbird strain carried only *stx*_2a_ genes, had a serotype of O17:H18, and belonged to ST6507.

### 3.2. Comparative pathogenomic analyses

**FIGURE 1:**
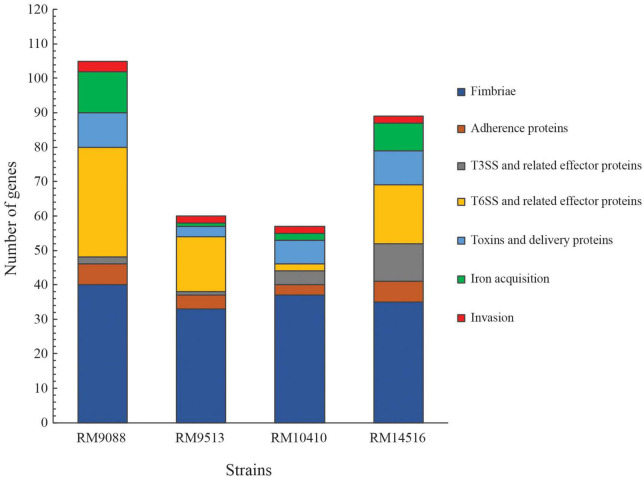
Detection of virulence genes in avian *E. coli* strains. The functional categories of the virulence genes are color-coded. The list of genes, their products and associated *E. coli* pathotypes are presented in Supplementary Table 1. Presence of each virulence factor in each strain was verified by BLASTn search of a database containing all avian strains in Geneious Prime^®^ software with a threshold of 65% for coverage and 70% for sequence identity.

A total of 313 genes related to bacterial adherence, biofilm formation, bacterial invasion, toxins/effectors production and delivery, and iron acquisition were examined systematically (Supplementary Table 1). Among the three *stx*-positive avian strains, 102, 55, and 87 virulence genes were detected in RM9088, RM10410, and RM14516, respectively, while 58 virulence genes were detected in *stx*-negative strain RM9513 (Figure 1). Strain RM9088 had the greatest number of genes related to fimbriae, Type VI Secretion System (T6SS), toxins and toxin delivery systems, and iron uptake and storage (Figure 1).

Like the EHEC prototype strain EDL933 and the nonpathogenic K-12 sub-strain MG1655, all four avian *E. coli* strains carried the genes encoding curli fimbriae, hemorrhagic *E. coli* pilus, and type I fimbriae. The *E. coli* common pilus genes were also identified in these avian strains although with a point deletion and an amber mutation in the *ecpC* of strain RM9088 and the *ecpD* of strain RM10410, respectively. In contrast, none of the avian strains carried the genes related to biosynthesis of the afimbrial adhesin AFA-I, the S fimbriae, or the aggregative adherence fimbriae that are commonly present on the large aggregative plasmid pAA in EAEC strains (Supplementary Table 1). Difference in fimbriae genes content was also revealed. For example, the *E. coli* laminin-binding fimbriae genes were detected in all crow strains, whereas the F1C fimbriae genes were present only in the blackbird strain RM14516 (Table 2). The crow strain RM9088 appeared to carry more fimbriae genes than any other avian strains examined. For example, homologs of adhesive K88 fimbriae genes were present on the pEHEC of RM9088; Homologs of colonization factor antigen I (CFA/I) genes were detected in the chromosome of RM9088 (Table 2). The crow strain RM10410 was the only one carrying homologs of P fimbriae genes although a single base deletion and an IS66 insertion were detected in *papH* and *papA*, respectively (Table 2).

**TABLE 2:** Genetic diversity in genes encoding fimbriae or adhesins in avian *E. coli* strains.

Fimbriae	Related genes	RM9088	RM9513	RM10410	RM14516
Adhesive fimbriae	*cfaA*	+	+	−	−
	*cfaB*	+	+	−	−
	*cfaC*	+	+	−	−
	*cfaD*	+	+	−	−
Adhesive fimbriae	*faeC*	+	−	−	−
	*faeD*	+	−	−	−
	*faeE*	+	−	−	−
	*faeF*	+	−	−	−
	*faeG*	+	−	−	−
	*faeH*	+	−	−	−
	*faeI*	+	−	−	−
	*faeJ*	+	−	−	−
Curli fimbriae	*cgsG*	+	+	+	+
	*cgsF*	+	+	+	+
	*cgsE*	+	+	+	+
	*cgsD*	+	+	+	+
	*csgB*	+	+	+	+
	*csgA*	+	+	+	+
	*csgC*	+	+	+	+
*E. coli* laminin-binding fimbriae	*elfA*	+	+	+	−
	*elfD*	+	+	+	−
	*elfC*	+	+	+	−
	*elfG*	+	+	+	−
*E. coli* common pilus (ECP)	*ecpR*	+	+	+	+
	*ecpA*	+	+	+	+
	*ecpB*	+	+	+	+
	*ecpC*	+*	+	+	+
	*ecpD*	+	+	+*	+
	*ecpE*	+	+	+	+
F1C fimbriae	*focA*	−	−	−	+
	*focI*	−	−	−	−
	*focC*	−	−	−	+
	*focD*	−	−	−	+
	*focF*	−	−	−	+
	*focG*	−	−	−	+
	*focH*	−	−	−	+
Hemorrhagic *E. coli* pilus (HCP)	*hcpA*	+	+	+	+
	*hcpB*	+	+	+	+
	*hcpC*	+	+	+	+
P fimbriae	*papX*	−	−	−	−
	*papG*	−	−	+	−
	*papF*	−	−	+	−
	*papE*	−	−	+	−
	*papK*	−	−	+	−
	*papJ*	−	−	+	−
	*papD*	−	−	+	−
	*papC*	−	−	+	−
	*papH*	−	−	+*	−
	*papA*	−	−	+*	−
	*papB*	−	−	+	−
	*papI*	−	−	+	−
Type I fimbriae	*fimB*	+	+	+	+
	*fimE*	+	+	+	+
	*fimA*	+	+	+	+
	*fimI*	+	+	+	+
	*fimC*	+	+	+	+
	*fimD*	+	+	+	+
	*fimF*	+	+	+	+
	*fimG*	+	+	+	+
	*fimH*	+	+	+	+
**Autotransporters/Adhesins**					
Antigen 43	*ag43*	+	−	+	+
Cah	*cah*	-	+	+	−
EaeH	*eaeH*	+	+	+*	+
Inverse autotransporter adhesin IatC	*eaeX/air*	−	−	−	+
EhaB, AIDA-I type	*ehaB*	+	+	+*	+
Porcine attaching-effacing protein	*paa*	+	−	−	−
UpaG adhesin	*upaG/ehaG*	+	+	−	+
UpaH	*upaH*	+	−	+	+

+, presence; −, absence; +*, gene is present but carrying a loss-of-function mutation as detailed in Supplementary Table 1.

Similarly, differences in adhesin genes profile were detected among the avian strains. The genes encoding intimin-like adhesin EaeH and an AIDA-1 type autotransporter EhaB were present in all avian strains; the *upaG* gene was detected in all strains except RM10410; and the genes *agn43* and *upaH* were detected in all strains except RM9513 (Table 2). Moreover, *cah*, encoding the autoaggregative protein Cah was detected in strains RM9513 and RM10410; *paa*, encoding a porcine attaching-effacing associated protein, was only detected in the crow strain RM9088; and *eaeX*, encoding an inverse autotransporter adhesin IatC, was only detected in the blackbird strain RM14516 (Table 2).

Subsequent examination of the genes related to the production and delivery of toxins and effectors revealed that, unlike the strain EDL933, none of the avian *E. coli* strains carried homologs of genes involved in biosynthesis of the Type III Secretion System (T3SS) apparatus (Supplementary Table 1). Consistently, no LEE island was identified in any of the avian strains. Among the 60 genes encoding T3SS effector proteins examined, homologs of 13 effector genes were identified in strain RM14516, while five effector genes were identified in other avian strains (Supplementary Table 1).

**FIGURE 2:**
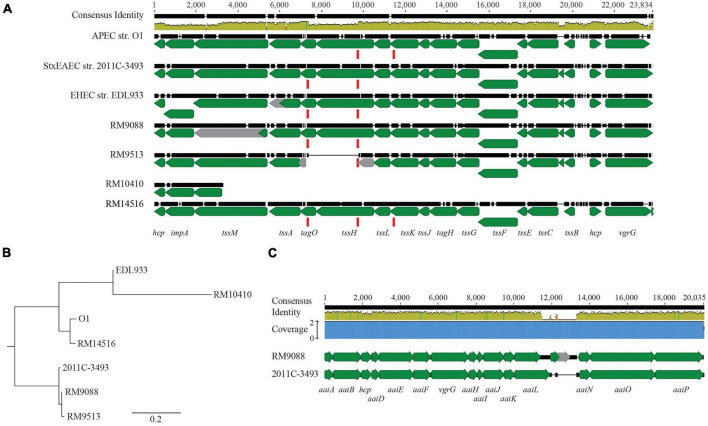
T6SSs in avian *E. coli* strains. **(A)** Genetic organization of T6SS-2 in the avian *E. coli* strains. The T6SS-2 genes in the avian strains were aligned and compared with the T6SS-2 genes in EHEC strain EDL933, APEC strain O1, and STEC strain 2011C-3493 using Clustal Omega in Geneious Prime^®^ software. Green arrows represent genes while gray arrows represent pseudo genes. The direct repeat (DR: CCGGCACCTG) (represented by the red block) identified in genes *tagO* and *tssH* was also present in gene *tssK* in the APEC strain O1 and the avian strain RM14516. The identity of the consensus sequence is color-coded (Green: 100% identity; Brown: at least 30% and under 100% identity; and Red: below 30% identity). **(B)** Phylogenetic analyses of T6SS-2. A consensus tree was constructed in Geneious Prime^®^ software using Geneious Tree Builder with following settings: Genetic Distance Model: Jukes-Cantor; Tree Build Method: Neighbor-Joining; Outgroup: No; Resampling Method: Bootstrap; Random Seed: 582,685; Number of Replicates: 10,000. **(C)** Comparative analyses of T6SS-3 in strain RM9088. Pairwise alignment of T6SS-3 genes in avian strain RM9088 with the T6SS-3 genes in STEC strain 2011C-3493 using Clustal Omega. Green arrows represent genes while grey arrows represent pseudo genes. Blue block represents the coverage. The identity of the consensus sequence is color-coded (Green: 100% identity; Brown: at least 30% and under 100% identity; and Red: below 30% identity).

The *E. coli* T6SS gene clusters are classified in three distinct phylogroups (T6SS-1 to T6SS-3) based on the gene organization and sequence similarities (Journet and Cascales, 2016), and among them, T6SS-1 and T6SS-2 are the most prevalent. Examining the three T6SS gene clusters revealed that, although homologs of T6SS-1 genes were not identified in any of the avian strains, homologs of T6SS-2 genes were identified in all strains (Figure 2A). A total of 18 genes in strain RM9088 showed homology to T6SS-2 genes and were clustered on a 23-Kb GI located upstream of the gene encoding a RHS repeating protein (locus tag: FRV13_02745). All genes were predicted to be functional except *tssM* (locus tag: FRV13_02665), which carried an amber mutation. A T6SS-2 gene cluster was also identified in strain RM9513, located upstream of the gene encoding a RHS repeating protein (locus tag: FS836_01975) (Figure 2A). However, unlike the T6SS-2 in strain RM9088, a large deletion (∼2.4 Kb) spanning from the coding sequence of *tagO* to the coding sequence of *tssH* was revealed in strain RM9513. This deletion appeared to be mediated by the recombination between the two direct repeats located within the coding sequence of *tagO* and *tssH*, respectively, based on sequence analyses (Figure 2A). All 17 T6SS-2 genes in strain RM14516 were predicted to be functional. In strain RM10410, a short DNA segment containing the T6SS genes *hcp*, *impA*, and a truncated *tssM* was identified, located at the same chromosomal locations as the T6SS-2 in other avian strains. Sequence alignment revealed that the T6SS-2 genes in strain RM14516 was highly similar to the T6SS-2 in APEC strain O1, while the T6SS-2 genes in both RM9088 and RM9513 were more closely related to T6SS-2 genes in the STEC O104:H4 outbreak strain 2011C-3493 (Figure 2B). Homologs of T6SS-3 genes were only revealed in strain RM9088, which were located on the plasmid pEHEC. High sequence similarity was detected for a total of 15 genes when compared to the T6SS-3 genes identified in the STEC O104:H4 outbreak strain 2011C-3419, regardless of an IS110 insertion sequence between the genes *aaiL* and *aaiN* in strain RM9088 (Figure 2C).

In addition to T3SS and T6SS, other secretion systems and autotransporters to deliver toxins or effector proteins were examined systematically. Among the 13 genes related to delivery of toxins or effector proteins, only a few were detected, including *cdiAB* in all four strains, *pic* in strain RM9088, *espI* in strain RM10410, and *espP* in strain RM14516 (Supplementary Table 1). Besides the *stx* genes and enterohemolysin genes described previously, the chromosome-borne hemolysin gene *hlyE* was detected in all avian strains, although a large deletion and an IS4-insertion in the coding sequencing of *hlyE* was detected in RM9088 and RM10410, respectively. Interestingly, the heat-labile toxin genes *eltAB* were detected in strain RM9088, implying that RM9088 carried a hybrid pathotype of STEC and ETEC.

### 3.3. Comparative analyses of Stx-prophages

**FIGURE 3:**
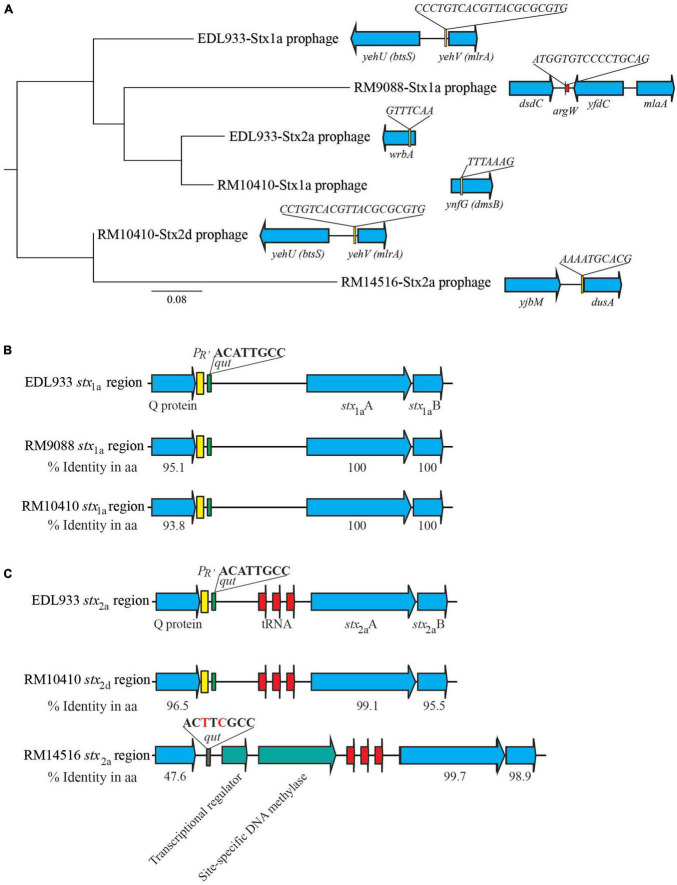
Sequence analyses of Stx-prophages in avian *E. coli* strains. **(A)** Phylogeny and chromosomal locations of Stx-prophages. The Stx-prophages and the putative integration sites were identified using PHASTER. Each Stx-prophage genome was extracted from the corresponding host genome. Sequences were aligned using Geneious Alignment and a consensus Neighbor-Joining tree was constructed in Geneious Prime^®^ Software as detailed in the Material and Methods section. The genome sequences of BP-933W, the Stx2a-prophage of STEC O157:H7 strain EDL933, and CP-933V, the Stx1a-prophages of the strain EDL933, were retrieved from GenBank under the accession number AE005174. The chromosomal locations of Stx-prophages are shown in the schematic maps. Genes are not drawn to scale. Red arrows indicate tRNA genes. **(B)** Sequence analyses of the *stx*_1_ regions. The percent identity represents the pairwise comparison of protein sequences with the corresponding protein in strain EDL933. Yellow blocks refer to the late promoter *P*_*R’*_ and the green blocks refer to the *qut* site. **(C)** Sequence analyses of the *stx*_2_ regions. The percent identity represents the pairwise comparison of protein sequences with the corresponding protein in strain EDL933. Red arrows indicate tRNA genes; yellow block refers to the late promoter *P*_*R’*_; green blocks refer to the *qut* site; and the gray block refers to an altered *qut* site.

Diverse integration sites for Stx-prophages were revealed in the avian STEC strains. The Stx1a-prophage in RM9088 was inserted within the gene *ompW*, the same insertion site utilized by the Stx2a-prophages in STEC O121:H19 strains (Carter et al., 2022b; Table 3). This integration site in strain EDL933 is occupied by the O-island #102. In RM10410, the Stx1a-prophage was inserted within the *ynfG* gene (also known as *dmsB*), while the Stx2d-prophage was inserted at the same site as the Stx1a-prophage in strain EDL933 (Table 3). The Stx2a-prophage in strain RM14516 is inserted within gene encoding the tRNA dihydrouridine synthase DusA (Figure 3A). Sequence comparison of the Stx-prophages in the avian strains with those in EDL933 revealed two clusters (Figure 3A). Both Stx1a-prophages in the crow strains RM9088 and RM10410 were grouped together with the Stx1a- and Stx2a-prophages in EDL933, while the Stx2d in the crow strain RM10410 displayed the highest sequence similarity with the Stx2a-prophage in the blackbird strain RM14516.

**TABLE 3:** Genomic characteristics and chromosomal locations of Stx-prophages in avian STEC strains.

Strains	Serotype	Stx-prophages^a^				
		*stx* genes	Chromosomal locations	Size (bp)	%GC	CDS
EDL933	O157:H7	*stx*_1a_	*btsS*–(2,966,157–3,015,072)–*mlrA*	48,916	52.0	64
		*stx*_2a_	wrbA*–(1,330,836–1,392,491)–wrbA*	61,663	49.4	69
RM9088	O109:H48	*stx*_1a_	*dsdC*–(4,690,253–4,731,904)–*argW*	41,652	49.6	73
RM10410	O113:H4	*stx*_1a_	ynfG*–(1,803,652–1,866,151)–ynfG*	62,500	49.6	89
		*stx*_2d_	*btsS*–(2,424,321–2,478,064)–*mlrA*	53,744	50.5	82
RM14516	O17:H18	*stx*_2a_	*yjbM*–(4,476,754–4,525,768)–*dusA*	49,015	52.3	70

^a^Prophages were initially identified by PHASTER and manually corrected by mapping the corresponding bordering regions in the genome of K-12 sub-strain MG1655. Genes represent the ones flanking the prophage genome.

*truncated genes.

The regulatory elements of the *stx*_1a_ in the crow strains RM9088 and RM10410, including antitermination protein Q, the late promoter *P*_*R’*_, and the *qut* site, were highly similar to those carried by the EDL933 Stx1a-prophage (Figure 3B). The regulatory elements of the *stx*_2d_ in the crow strain RM10410 was highly similar to those carried by the EDL933 Stx2a-prophage (Figure 3C). However, this region exhibited a large sequence divergence in the blackbird strain RM14516. Specifically, the Stx2a-prophage in strain RM14516 encoded an antitermination protein exhibiting 47.6% sequence identity with the Q protein encoded by EDL933 Stx2a-prophage, carried an altered *qut* site, and lacked the late promoter *P*_*R’*_. Moreover, there were two CDSs located between the coding sequences of the antitermination protein gene and the *stx*_2a_.

### 3.4. Comparative analyses of PAIs and GIs

**FIGURE 4:**
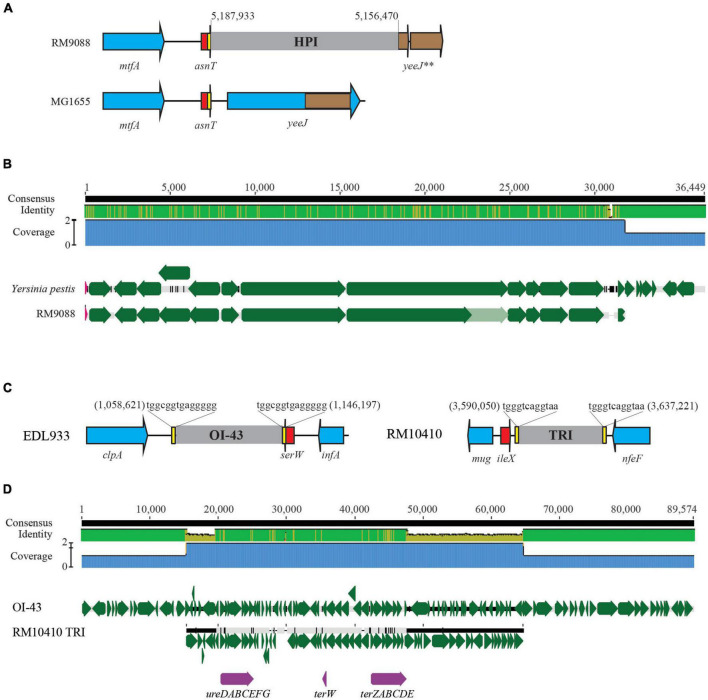
Comparative analysis of HPI and TRI in avian STEC strains. **(A)** Chromosomal locations of the HPI in strain RM9088. This insertion site is unoccupied in nonpathogenic *E. coli* strain MG1655 and in other avian *E. coli* strains examined in this study. Numbers indicate the chromosomal position of HPI in strain RM9088. Red arrows indicate the tRNA genes; Blue arrows indicate the bordering genes; Brown arrows are pseudogenes. Brown block within the gene *yeeJ* refers to the homologous region of the pseudo genes bordering HPI. Genes are not drawn to scale. **(B)** Pairwise alignment of *Yersinia pestis* HPI sequence with the RM9088 HPI sequence. The identity of consensus is colored coded (Green: 100% identity; Brown: at least 30% and under 100% identity; Red: below 30% identity). Green arrows represent genes located on the HPIs. The genes carrying frame-shift mutations are indicated by the light green arrows. **(C)** Chromosomal locations of the TRI in strain RM10410 in comparison with the OI-43 in strain EDL933. The chromosomal locations of TRI and OI-43 are indicated by the numbers referencing the start and the end of the bordering Direct Repeats (DRs). Red arrows represent tRNA genes; Yellow blocks refer to the DRs; Blue arrows represent the bordering genes; Grey blocks refer to the TRIs. Genes are not drawn to scale. **(D)** Pairwise alignment of EDL933 OI-43 with the TRI in strain RM10410. The identity of consensus is color-coded: Green: 100% identity; Brown: at least 30% and under 100% identity; Red: below 30% identity. Green arrows represent the genes on the TRIs whereas purple arrows represent the urease gene cluster and the tellurite resistance genes.

A 13-Kb AFI was present in strain RM9088, carrying the genes related to acid resistance and the genes encoding the efflux pump. This AFI was nearly identical to the AFI in *E. coli* strain MG1655 (% Identity, 99.1) (Table 4). The complete LAA is about 81 Kb and carries 89 CDSs as previously reported in STEC O91:H21 strain B2F1 (Montero et al., 2017). The LAA virulence genes are distributed in the four modules, including *sisA* and *hes* on module I, *iha* and *lesP* on module II, *pagC*, *tpsA* and *tpsB* on module III, and *agn43* on module IV. A complete LAA was not detected in RM9088, but homologs of module I and module IV were identified (Table 4). The module I carried the genes *atoSC* and *atoDAB*, related to acetoacetate metabolism, and *atoE*, a short chain fatty acid transporter gene. Neither of the two virulence genes (*sisA* and *hes*) were present in module I and no homologs of *sisA* or *hes* were identified after searching the entire RM9088 genome. The LAA module IV was similar in length when compared to the reference strain B2F1, exhibiting over 74% sequence identity, and carrying the virulence gene *agn43*. On the identification of additional PAIs in strain RM9088, no homologs of OI-57, OI-122, and TRI were revealed, however, a complete HPI was detected. In *Y. pestis*, the 36-Kb HPI is located on a 102-Kb mobile GI adjacent to the tRNA gene *asnT*, and harbors genes involved in iron uptake (Buchrieser et al., 1998; Carniel, 1999). The 32-Kb HPI in strain RM9088 was found also integrated downstream of the tRNA gene *asnT* (Figure 4A). Furthermore, this HPI exhibited 99% sequence identity with the HPI in *Y. pestis* and carried all genes required for the biosynthesis, transport, and regulation of the siderophore yersiniabactin (Figure 4B). The putative integration site for HPI is conserved in the crow strains RM9513 and RM10410, and in several STEC strains belonging to the clinically relevant serotypes including STEC O104:H4 and STEC O26:H11 (Data not shown).

**TABLE 4:** Genetic features of Pathogenicity Islands (PAIs) and Genomic Islands (GIs) in avian *E. coli* strains.

Strains	AFI	HPI	LAA			OI-57	TRI^a^	
	Locations [Size (bp) / %GC]	Locations [Size (bp) / %GC]	Locations [Size (bp) / %GC]			Locations [Size (bp) / %GC]	Locations [Size (bp) / %GC]	
MG1655	3,653,961–3,667,580 (13,620 / 46.0)	ND	Module I: 2,320,014–2,327,293 (7,280 / 50.9)		Module IV: 2,070,659–2,078,037 (7,379 / 54.8)	ND	ND	
EDL933	4,454,268–4,476,943 (22,676 / 47.6)	ND	Segments of Modules I, II, III, and IV were detected on the two TRIs.			1,849,324–1,929,825 (80,502 / 51.4)	1,058,635–1,146,183 (87,549 / 48.0)	1,454,242–1,541,789 (87,548 / 48.0)
RM9088	3,417,899–3,431,516 (13,618 / 45.9)	5,187,933–5,156,470 (31,464 / 57.7)	Module I: 4,878,390–4,871,110 (7,281 / 50.9)		Module IV: 3,993,364 – 4,016,157 (22,794 / 52.3)	ND	ND	
RM9513	3,664,504–3,678,124 (13,621 / 46.0)	ND	Module I: 2,318,003–2,325,282 (7,280 / 50.9)		Module IV: 2,077,408–2,084,213 (6,806 / 55.8)	1,423,621–1,442,297 (18,677 / 44.2)	ND	
RM10410	4,078,840–4,092,459 (13,620 / 46.0)	ND	Module I: 2,581,875–2,589,154 (7,280 / 50.9)	Module II + III: 3,027,004–3,078,404 (51,401 / 48.2)	Module IV: 4,295,579–4,312,440 (16,862 / 52.2); 4,845,765–4,872,929 (27,165 / 49.4)	ND	3,590,062–3,637,209 (47,148 / 50.3)	
RM14516	3,814,404–3,837,034 (22,631 / 47.8)	ND	Module I: 2,405,119–2,412,398 (7,280 / 50.9)	Module III: 4,643,912–4,658,535 (14,624 / 53.4)	Module IV: 4,626,787–4,639,584 (12,798 / 52.1)	ND	ND	

^a^The TRIs islands in strain EDL933 correspond to O-island #43 and #48 as reported previously (Perna et al., 2001). ND: not detected.

As observed for strain RM9088, strain RM9513 carried an AFI highly similar to the AFI in strain MG1655, and an incomplete LAA with gene homologs in the module I and IV fragments, located apart on the chromosome (Table 4). The LAA module I fragment carried the genes related to acetoacetate metabolism but lacked the virulence genes *sisA* and *hes*. The module IV carried the autotransporter gene *cah*, a homologue of *agn43* (Supplementary Table 1). No homologs of LAA module II or III were identified in RM9513. Unlike strain RM9088, strain RM9513 carried an incomplete OI-57, exhibiting sequence similarity with a 20-Kb DNA segment in the OI-57 in strain EDL933. The complete OI-57 is about 80 Kb, carrying virulence genes *adfO* (*paa*) and *ckf*, and is associated with the highly pathogenic seropathotypes A and B (Imamovic et al., 2010). The virulence gene *ckf* was present in the OI-57 in strain RM9513.

The AFI in strain RM10410 was highly similar to the AFIs in strains MG1655, RM9088, and RM9513 (Table 4). However, unlike the crow strains RM9088 or RM9513, a 47-Kb TRI was detected in RM10410, which was located adjacent to the tRNA gene *ileX* and contained the genes conferring resistance to tellurite and the urease gene cluster (Figures 4C, D). Similar to the TRIs in EHEC strain EDL933, two 12-bp DRs were identified flanking the TRI in strain RM10410 (Figure 4C). Homologs of LAA module I, II/III, and IV were detected at three separate locations on the RM10410 chromosome (Table 4). The module I was highly similar to the LAA module I in strains MG1655, RM9088, and RM9513. However, unlike the above strains, both virulence genes (*sisA* and *hes*) were detected in the RM10410 genome but located outside of module I (*sisA*: 4,940,577–4,939,534; *hes*: 3,443,911–3,443,165). Homologs of LAA module II and III were located adjacently, containing all virulence genes on the LAA module II and III in the reference strain B2F1, including *iha* (locus tag: FS611_15505) and *lesP* (locus tag: FS611_15475) on the module II, and *pagC* (locus tag: FS611_15370), *tpsA* (locus tag: FS611_15350), and *tpsB* (locus tag: FS611_15330) on the module III. Additionally, two homologs of LAA module IV were detected in strain RM10410 (Table 4), one contained virulence gene *agn43* (locus tag: FS611_21665) while the other one contained the gene *cah* (locus tag: FS611_24255).

Among the avian strains examined, only the blackbird strain RM14516 harbored a 22-Kb AFI highly similar to the one in strain EDL933 (Table 4), in which, the iron acquisition genes *chuSA* and *chuTWXYU* were inserted between the genes encoding a transcription regulator DctR (locus tag: FS841_18470) and a transport ATPase YhiD (locus tag: FS841_18520). Homologs of LAA module I, III, and IV were also detected in strain RM14516 but were located in different regions on the chromosome. The LAA module I carried the genes related to acetoacetate metabolism but lacked the virulence genes *sisA* and *hes*, similarly to the LAA module I in strains RM9088, RM9513, and MG1655 (Table 4). Although no homolog of LAA module II was identified in strain RM14516, the virulence genes *iha* and *lesP* were present on the plasmid pEHEC. The module III was highly similar to the LAA module III in the reference strain B2F1 (% identity, >90), containing virulence gene *tpsA* and *tpsB*. No homolog of *pagC* was revealed in the genome of RM14516. The LAA module IV in strain RM14516 contained virulence gene *agn43* and exhibited 76% sequence identity with the LAA module IV in the reference strain B2F1. Although a complete SE-PAI was not detected in strain RM14516, the virulence genes *subAB* (locus tags: FS841_23955 and FS841_23960), encoding AB_5_ subtilase cytotoxin, were present on the plasmid pEHEC.

### 3.5. Biofilm formation in avian *E. coli* strains

**FIGURE 5:**
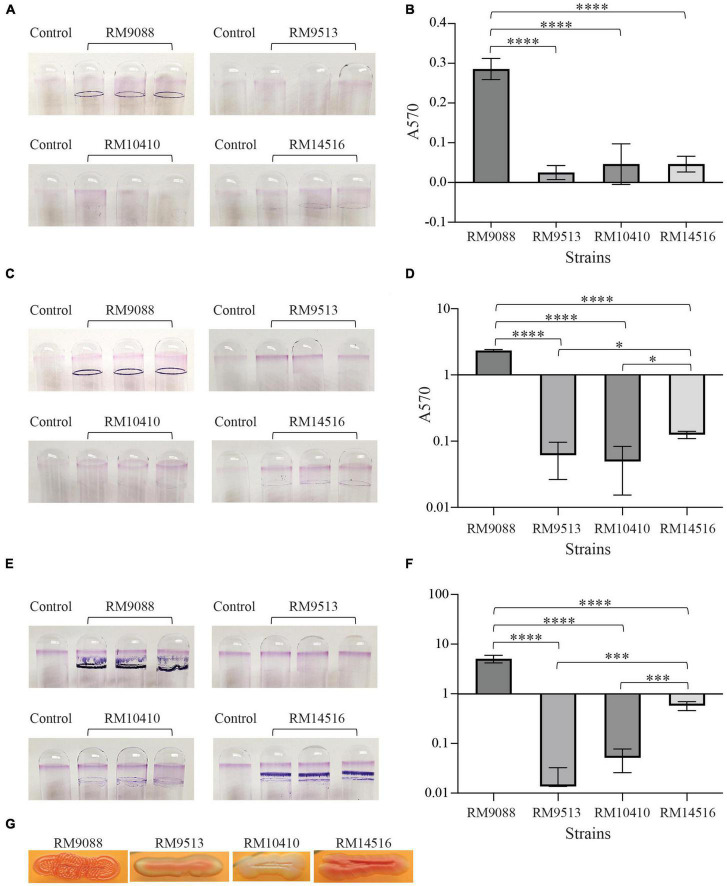
Biofilms by avian *E. coli* strains on glass surfaces. Crystal violet staining the biofilm biomass on the glass culture tubes under the static condition for 24 h **(A)**, 48 h **(C)**, and 120 h **(E)**, and quantitative assay of biofilms under the static condition for 24 h **(B)**, 48 h **(D)**, and 120 h **(F)**. Each data set represents the mean absorbance and SD from at least three biological replicates. The differences in the means between the two given strains are indicated with the adjusted *P*-values of Tukey’s multiple comparisons test following the One-way ANOVA test (*P ≤ 0.05, ***P ≤ 0.001; and ****P ≤ 0.0001). The biofilms of strain RM14516 following 48 h and 120 h incubation were significantly greater than that of the strains RM9513 and RM10410 only when the three strains (RM14516, RM10410, and RM9513) were compared in the statistical tests. **(G)** Production of curli fimbriae on CRI plates following incubation at 26°C for 48 h.

Biofilm formation by *E. coli* avian strains was evaluated quantitatively. Following 24 h incubation, a great amount of surface-associated biomass, exemplified as a strong ring on the glass surface, was observed on all tubes inoculated with strain RM9088 (Figure 5A). Consistently, the biofilm of RM9088 was significantly greater than that of any other strains examined (One-way ANOVA test followed by Tukey’s test, adjust *P* < 0.0001) (Figure 5B). Although a visible dye ring was observed on glass tubes inoculated with strain RM14516, the biofilm was not significantly greater than that of strains RM9513 or RM10410. No visible rings were observed for tubes inoculated with either RM9513 or RM10410. Following 48 h of incubation, biofilms of strain RM9088 increased significantly compared with the biofilms at 24 h (One-way ANOVA test followed by Tukey’s test, adjust *P* = 0.0064) and was significantly greater than that of any other strains examined (One-way ANOVA test followed by Tukey’s multiple comparisons test, adjust *P* < 0.0001) (Figure 5D). Biofilms of strain RM14516 at 48 h were significantly greater than that of strains RM9513 or RM10410 when the ANOVA test was performed for strains RM14516, RM9513, and RM10410 (Tukey’s test, adjust *P* < 0.05). No visible rings were observed for tubes inoculated with strain RM9513 or RM10410 following either 24 h or 48 h incubation (Figures 5A, C). The levels of biofilm by both strains RM9088 and RM14516 continued to increase when the incubation time was extended to 120 h (Figure 5E). At 120 h, the biofilms of strain RM9088 were 17.7- and 2.2-fold larger in size when compared to the biofilms detected at 24 and 48 h, respectively. Moreover, the levels of biofilm by strain RM14516 were 12.4- and 4.6-fold higher than those detected at 24 and 48 h, respectively (Figure 5F). In contrast, no biofilms were detected for strains RM9513 or RM10410 even when incubation time was increased to 120 h.

Because all avian strains harbored intact curli genes, production of curli fimbriae were examined. Strains RM9088 and RM14516 were found to be a strong and moderate curli producer as colonies exhibited a dark red and red color on CRI, respectively. By contrast, RM9513 was a weak curli producer and RM10410 was a curli-deficient strain as colonies grown on CRI plates appeared pink and white, respectively (Figure 5G).

### 3.6. Cytotoxicity of avian *E. coli* strains

**FIGURE 6:**
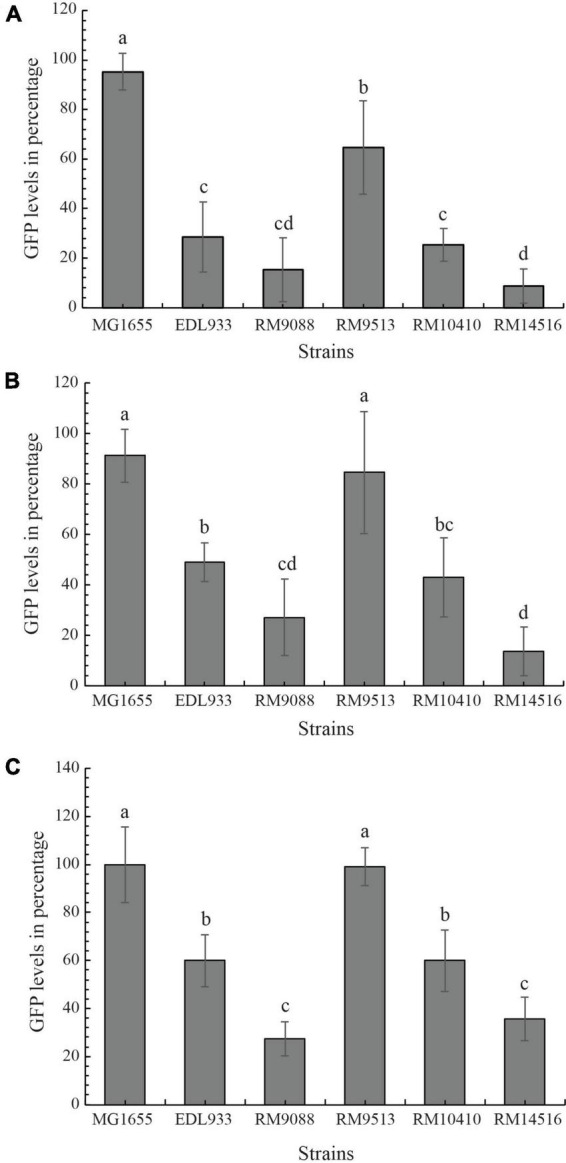
Cytotoxicity of avian *E. coli* strains. The Stx-mediated cytotoxicity in cultured Vero cell line, Vero-d2EGFP. Avian *E. coli* strains were examined in comparison with K-12 sub-strain MG1655 (negative control) and STEC O157:H7 strain EDL933 (positive control) using 1:10 diluted **(A)**, 1:100 diluted **(B)**, and 1:1000 diluted culture supernatants **(C)**. High GFP levels represent low Stx cytotoxicity. The cytotoxicity levels in the Vero-d2EGFP cells when testing cell-free culture supernatants from the *E. coli* strains were examined with a One-way ANOVA test followed by a Tukey-HSD *post hoc* analysis in R version 4.2. *E. coli* strains with statistically significant differences in the Vero-d2EGFP cell cytotoxicity levels (*P*-value < 0.05) were assigned different lower-case letters.

The Stx-mediated cytotoxicity of the avian strains was assessed by using the mammalian host Vero-d2EGFP cells and bacterial cell-free culture supernatants. Incubation with high concentration of active Stx resulted in low levels of fluorescence in the host Vero-d2EGFP cells. The detected levels of cytotoxicity in the examined avian strains were compared to those levels detected when testing Stx-negative *E. coli* strain MG1655 and the Stx-positive EHEC prototype strain EDL933. When testing a 10-fold dilution of culture supernatant from avian strain RM10410 (*stx*_1a_ + *stx*_2d_), the cytotoxicity level was comparable to the EHEC strain EDL933 (*stx*_1a_ + *stx*_2a_) and was significantly higher in Stx activity than the negative control strain MG1655 and the *stx*-negative crow strain RM9513 (Figure 6A). Interestingly, the greatest cytotoxicity was detected when testing culture supernatants from strains RM9088 (*stx*_1a_) and RM14516 (*stx*_2a_), which both were higher than that of the EHEC strain EDL933. A similar result was observed when the Vero-d2EGFP cells were intoxicated with the 100-fold dilution of the Stx-containing supernatants (Figure 6B). When the Vero-d2EGFP cells were intoxicated with the 1000-fold dilution of the Stx-containing supernatants, the avian strain RM9088 exhibited greater cytotoxicity than strain RM14516, which both were significantly greater than any of other strains examined. The avian strain RM10410 displayed similar cytotoxicity levels as the strain EDL933, both of which were significantly higher than the negative control strain MG1655 and the *stx*-negative avian strain RM9513 (Figure 6C).

## 4. Discussion

Rapid identification of foodborne pathogens that have a potential to cause severe human disease remains one of the biggest challenges in food safety. Considering the rapid virulence evolution in STEC, strains with the potential to persist and cause severe diseases in humans are likely emerging in diverse ecological niches. To date, there are over 470 serotypes of STEC reported and over 200 of them have been linked to enteric infections in humans (Valilis et al., 2018; FAO/WHO STEC Expert Group, 2019). STEC strains with enhanced pathogenicity potential and persistence could emerge from any of the serotypes considering that many genes encoding virulence traits and virulence related traits, such as adherence and biofilm formation, are often associated with MGEs. In fact, it has been recommended by The National Advisory Committee on Microbiological Criteria for Foods that virulence genes profile or virulence genes repertoire, rather than serotype is much more meaningful in predicting the pathogenicity potential in uncharacterized STEC strains (National Advisory Committee on Microbiological Criteria for Foods, 2019). Built on the current knowledge about the pathogenesis of STEC and the epidemiological data, a combination of Stx subtypes and the adherence capacity has been used to predict the pathogenicity potential of STEC. Because the subtypes Stx1a, Stx2a, and Stx2d are most frequently implicated in causing severe human illness including HUS (Scheutz, 2014), any STEC strains that possess one of the above Stx subtypes and also express intimin adhesin, such as in EHEC strains, or aggregative fimbriae, such as in StxEAEC strains, are considered high-risk STEC due to the likelihood of causing severe human disease.

Strain RM9088, isolated from a crow, displayed the greatest cytotoxicity and was also the best biofilm producer among the strains examined. Unlike strain EDL933, RM9088 carried only one set of *stx* genes (*stx*_1a_). Although the Stx1a-prophage in strain RM9088 differed considerably from the Stx1a-prophage in strain EDL933, the regulatory region of *stx*_1a_ including the late promoter *P*_*R’*_, the *cis* element *qut*, and the Q protein in the two Stx1a-prophages were highly similar among both strains. This finding would imply a similar regulation mechanism governing the expression of *stx*_1a_, as supported by quantification of Stx1a production in strains RM9088 and EDL933. Under the non-inducing conditions, there was no significant difference in Stx1a production between EDL933 and RM9088 (Data not shown). The higher cytotoxicity of RM9088 compared to EDL933 may be attributed to other virulence factors present in this strain. Besides Stx and enterohemolysin, strain RM9088 carried genes encoding heat-labile enterotoxin (LT) (*eltA*, locus tag: FRV13_09375; *eltB*, locus tag: FRV13_09380), a signature toxin produced mainly in ETEC strains (Nataro and Kaper, 1998). Therefore, strain RM9088 truly is a hybrid of STEC and ETEC. LT is a virulence factor responsible for causing diarrhea in both humans and animals. Recent studies revealed that LT could enhance bacterial adherence and subsequent intestinal colonization of the pathogen (Duan et al., 2019). RM9088 also carried a gene (locus tag: FRV13_00070) encoding a heat-stable enterotoxin, and two genes encoding homologs of subtilase cytotoxin subunit B (locus tags: FRV13_08400 and FRV13_09355). Furthermore, strain RM9088 harbored *cdiAB*, encoding contact-dependent inhibition (CDI) system, two sets of T6SS genes similar to the STEC O104:H4 strains linked to the 2011 large outbreak of enterohemorrhagic infection in Europe (Ahmed et al., 2012), and two genes encoding homologs of the serine protease autotransporter toxin Pic. CDI, a type V delivery system, uses a long β-helical cell surface protein to contact the target cells and deliver a growth inhibition signal in target cells (Ikryannikova et al., 2020). CDI is widespread in gram-negative bacteria including several important human pathogens and plays a role in interbacterial competition and communication (Cuthbert et al., 2022). A recent study demonstrated that CDI was a key virulence determinant in the human pathogen *Pseudomonas aeruginosa* due to its contribution to toxicity in cultured mammalian cells and lethality in mouse model system due to bacteremia (Allen et al., 2020). The CdiA in strain RM9088 encodes a contact-dependent inhibition effector tRNA nuclease, thus the CDI in RM9088 likely functions in a similar fashion as the CDIs in UPEC strain 536 and EHEC strain EC869 (Diner et al., 2012; Wang et al., 2022). Like CDI system, T6SS can directly target both prokaryotic and eukaryotic cells and contribute to interbacterial competition, nutrient acquisition, and pathogenesis (Navarro-Garcia et al., 2019; Wood et al., 2020). The serine protease autotransporter toxin Pic was initially identified in EAEC and *Shigella flexneri* strains (Henderson et al., 1999). Pic has been shown to promote intestinal colonization and growth of the enteric pathogens in the presence of mucin and is responsible for the EAEC-induced hypersecretion of mucus as well as the mucoid diarrhea induced by *S. flexneri* (Harrington et al., 2009; Navarro-Garcia et al., 2010). Both *pic* genes in strain RM9088 were plasmid-borne and each encoded an autotransporter toxin distantly related to the EAEC Pic. When compared to the EAEC Pic, the Pic encoded by the large virulence plasmid pEHEC (locus tag: FRV13_00240) in RM9088 exhibited 35% amino acids sequence identity while the Pic encoded by the second plasmid (locus tag: FRV13_01160) exhibited 42% amino acids sequence identity.

Strain RM9088 also harbored numerous genes encoding adhesins or adhesive fimbriae. The adherence factors identified in strain RM9088, but not in the other examined avian strains included the adhesin protein Paa and fimbrial adhesin K88 (Table 2; Supplementary Table 1). The *paa* gene, originally identified in a porcine enteropathogenic strain, is present in both EHEC and ETEC strains (An et al., 1999). Paa contributes to the formation of A/E lesions and thus is an important virulence factor in various *E. coli* pathotypes (An et al., 1999; Batisson et al., 2003). Paa in strain RM9088 (locus tag: FRV13_09320) is nearly identical to the Paa in several EHEC strains, implying a potential role in the pathogenesis of RM9088. The K88 fimbriae are mainly expressed in ETEC strains, mediating the binding of ETEC cells to epithelial cells in the pig small intestine (Sellwood, 1980; Middeldorp and Witholt, 1981). As observed for ETEC strains, the K88 fimbriae genes (*faeCDEFGHIJ*) in strain RM9088 were located on the large virulence plasmid pEHEC, exhibiting > 84% nucleotides sequence identity with the K88 fimbriae genes in porcine ETEC strain UMNK88. In addition to the K88 fimbriae, strain RM9088 harbors *cfaABCD* genes, encoding the CFA/I, the first known human-specific colonization factor in ETEC (Evans et al., 1978) and also present in other *E. coli* pathotypes and diverse non-O157 STEC strains (Klemm, 1979; Hacker, 1992). The *cfaABCD* genes in strain RM9088 displayed > 92% nucleotides sequence identity with the *cfa* genes in STEC strains belonging to clinically relevant serotypes O104:H4, O111:H6, O26:H11, O103:H2, and O121:H19. One possible explanation for the greatest ability in biofilm formation by strain RM9088 could be a combinational effect of multiple adherence factors. Strain RM9088 harbored genes encoding curli fimbriae, laminin-binding fimbriae, and the type I fimbriae. Under the experimental conditions to examine biofilm formation in this study, RM9088 produced curli fimbriae, an important adherence factor to attach to abiotic surfaces and to plant surfaces (Carter et al., 2016, 2019a).

Strain RM14516, recovered from a blackbird, exhibited greater cytotoxicity than the EHEC strain EDL933 when testing the various culture supernatant dilutions. Unlike EDL933, strain RM14516 harbored only *stx*_2a_ genes located on a 49-Kb prophage that showed sequence similarity to *Escherichia* phage 1720a-02 (GenBank accession number, KF030445) and prophages in STEC clinical isolate 89-3506 (GenBank accession number CP027520). In strain RM14516, the regulatory region of *stx*_2a_ genes, comprised of the DNA segment from the coding sequence of antitermination Q protein gene to the coding sequence of *stx*_2a_, differed largely from the corresponding region in strain EDL933, but exhibited high similarity (>98% identity) with the one in STEC O145:H28 strain RM13516, the outbreak strain linked to the 2017 ice cream associated outbreak in Belgium (Carter et al., 2021). Interestingly, under non-inducing conditions, strain RM14516 produced a greater amount of Stx2a than strain EDL933 (Data not shown). Besides Stx, RM14516 harbored both chromosome-borne hemolysin genes *hlyE* and the plasmid encoded enterohemolysin genes *hlyDBAC*. Although no LEE was identified in strain RM14516, a total of 13 genes homologs encoding the T3SS secreted effectors in EDL933 were identified in the genome of RM14516. Among the avian STEC strains examined, RM14516 was the only one harboring *espP*, encoding an extracellular serine protease that is capable of cleaving pepsin A and human coagulation factor V (Brunder et al., 1997) as well as the plasmid borne *subAB* (locus tags: FS841_23955 and FS841_23960), encoding AB_5_ subtilase cytotoxin, an important virulence factor in non-LEE STEC strains (Michelacci et al., 2013). Among the adherence factors examined, strain RM14516 was the only one carrying the inverse autotransporter adhesin gene (Locus tag: FS841_19375), which was identical to the inverse autotransporter adhesin IatC (GenBank Accession # WP_265468605.1). Previous studies demonstrated that the inverse autotransporters played a role in biofilm formation in *E. coli* and contributed to biofilm formation and virulence in *Yersinia ruckeri* (Martinez-Gil et al., 2017; Wrobel et al., 2020). Strain RM14516 was also the only one carrying the genes related to biogenesis of F1C fimbriae. Reports documented the F1C fimbriae to be mainly expressed by the UPEC strains along with type I fimbriae and P fimbriae for mediating pathogen colonization of the urinary tract (Riegman et al., 1990). F1C fimbriae have also been shown to contribute to biofilm formation and intestinal colonization in the *E. coli* commensal strain Nissle 1917 (Lasaro et al., 2009). Under the experimental conditions examined in this study, strain RM14516 was a moderate biofilm producer. Further studies are needed to elucidate if other fimbriae including F1C are involved in biofilm formation and pathogenesis of RM14516 using *in vivo* systems.

Strain RM10410, recovered from a crow, displayed a comparable cytotoxicity when compared to the EHEC strain EDL933 under all conditions examined. Unlike EDL933, strain RM10410 harbored the genes encoding Stx2d, a Stx subtype known to be activated by the intestinal mucus to become more toxic in host cells (Melton-Celsa et al., 1996). It is possible that the cytotoxicity of RM10410 could be potentially greater than strain EDL933 during mammalian host infection. The 54-Kb Stx2d-encoding prophage in RM10410 was inserted at the same chromosomal site as the Stx1a-encoding prophage in strain EDL933. Although the two prophages have low sequence similarity, the regulatory regions of *stx*_2a_ and *stx*_2d_ were nearly identical, potentially suggesting that a similar mechanism may govern the expression of both Stx subtypes. Other toxin genes present in strain RM10410 included *espI* (Locus tag: FS611_15475) and *hlyE*, encoding the EspI serine protease autotransporter and hemolysin, respectively. EspI, like EspP, belongs to the SPATE autotransporter family and contributes to EHEC and EPEC pathogenicity via proteolytic cleavage and inactivation of host proteins during infection (Mundy et al., 2004). The *espI* gene has been found to be widespread in EAEC strains (Andrade et al., 2017). Finally, although strain RM10140 carried intact curli genes, no curli fimbriae were detected under the experimental condition examined in this study, implying regulatory silence of curli production in this strain. Consistently, strain RM10410 produced very little biofilms under all conditions examined (Carter et al., 2011, 2016).

Current knowledge about the pathogenesis of STEC is largely limited to strains of several predominant groups such as O157:H7, StxEAEC O104:H4, and the “Big Six” non-O157 serogroups. In recent years, non-O157:H7 STEC serotypes associated with foodborne outbreaks and human infections have increased (Valilis et al., 2018), thus information about virulence and pathogenesis of STEC strains in diverse ecological niches is desired for the development of effective mitigation strategies. Wildlife has been recognized as a primary source of emerging infectious diseases, and these animal reservoirs can harbor uncommon or uncharacterized serotypes (Kim et al., 2020; Ray and Singh, 2022). Although all STEC strains have the potential to pose health risks, acquisition of certain virulence traits would promote the emergence of strains with a high-risk to human health. Adherence factors and toxins are two critical traits in STEC pathogenicity. During infection, adherence is the first step for a pathogen to establish colonization. Strong adherence often implies an efficient ability for surface attachment and biofilm formation, which would lead to enhanced survival and persistence in various environments. Our study revealed that the three avian STEC strains, recovered from a major agricultural region in California and belonging to uncommon serotypes O109:H48, O113:H4, and O17:H18, exhibited higher (RM9088 and RM14516) or comparable (RM10410) cytotoxicity with the EHEC prototype strain EDL933. Furthermore, strains RM9088 and RM14516 carried numerous genes encoding adhesins and adhesive fimbriae and produced a greater amount of biofilm under the experimental conditions examined, suggesting that these avian STEC strains likely have increased potential to cause severe human disease. Based on our comparative pathogenomic analyses, the virulence genes repertoire of strain RM9088 was a perfect example of “mix and match” virulence genes from different enteric pathogens. First, strain RM9088 appeared to be a hybrid of STEC-ETEC as it harbored genes encoding Stx and LT, signature toxins of STEC and ETEC, respectively. This hybrid pathotype was further supported by the fact that strain RM9088 carried genes encoding protein adhesion Paa and fimbrial adhesin K88, which both are commonly expressed in ETEC strains. Secondly, strain RM9088 possessed an array of genes encoding diverse toxins, toxin delivery systems, autotransporters, protein adhesins, and fimbrial adhesins that are found in different *E. coli* pathotypes, including EAEC, EPEC, ETEC, and EHEC, as well as in *Shigella* spp. Acquisition of HPI, a PAI associated with hyper-pathogenic *Yersinia* strains, implied an enhanced fitness trait for strain RM9088 when exposed to iron-limiting conditions including in human and animal hosts. In summary, our study revealed novel combinations of virulence factors in avian STEC strains and emergence of “high-risk” (increased likelihood to cause severe human disease) STEC strains in pre-harvest environments. The findings in this study support the notion that pathogenesis of STEC is a complex process and diverse mechanisms exist for governing pathogenicity of STEC. Comparative pathogenomic analysis is thus a powerful tool for assessing the pathogenicity and health risk of uncharacterized STEC strains.

## Data availability statement

The datasets presented in this study can be found in online repositories. The names of the repository/repositories and accession number(s) can be found in the article/Supplementary material.

## Ethics statement

Wildlife Sampling at all locations was approved under a set of California Department of Fish and Game (CDFG) Scientific Collection Permits issued to USDA Wildlife Services and CDFG personnel contracted to collect the samples and ship to USDA in Albany, California. Additionally, a federal permit with the U.S. Fish and Wildlife Services was obtained for sampling of geese, crows, and blackbirds. The wildlife sampling was conducted through a contract with state and federal wildlife agencies using their standard protocols. The study was conducted in accordance with the local legislation and institutional requirements.

## Author contributions

MQC: conceptualization, writing—original draft preparation, and project administration. AP, NL, DC, MC, BQ, XH, and MQC: methodology. AP: software. AP, NL, BQ, DC, and XH: data curation. BQ, MC, XH, and MQC: writing—review and editing and funding acquisition. All authors contributed to the article and approved the submitted version.
