# Supplementary material for: Pathogenicity assessment of Shiga toxin-producing Escherichia coli strains isolated from wild birds in a major agricultural region in California
Source: Front Microbiol. 2023 Sep 26;14:1214081. doi: 10.3389/fmicb.2023.1214081 (PMC10562709; doi:10.3389/fmicb.2023.1214081)
Supplement: Supplementary file 1 [file Data_Sheet_1.PDF]

Supplementary Table 1. Distribution of *E. coli* virulence genes in avian *E. coli* strains

Virulence Factor Catagories	Virulence factors	Related genes	Reference Genes		RM9088	RM9513	RM10410	RM14516
			Reference strain (GenBank accession number)	Locus tag				
**Adherence and Biofilm/Fimbriae**	**Aggregative adherence fimbriae**	*aafB*	EAEC str. 042 plasmid pAA (NC_017627.1)	EC042_RS26385	0	0	0	0
		aafC		EC042_RS26390	0	0	0	0
		aafD		EC042_RS26460	0	0	0	0
		aafA		EC042_RS26465	0	0	0	0
		aggD	StxEAEC str. 2011C-3493 plasmid pAA-EA11 (NC_018666.1)	O3K_RS26240	0	0	0	0
		aggC		O3K_RS26235	0	0	0	0
		aggB		O3K_RS26230	0	0	0	0
		aggA		O3K_RS26225	0	0	0	0
		aggR		O3K_RS26145	0	0	0	0
	**Adhesive fimbriae**	*cfaA*	EHEC str. 12009 (NC_013353)	ECO103_RS20275	1	1	0	0
		cfaB		ECO103_RS20270	1	1	0	0
		cfaC		ECO103_RS20265	1	1	0	0
		cfaD/cfaE		ECO103_RS20260	1	1	0	0
		faeC	ETEC str. UMNK88 pUMNK88_K88 (NC_017639.1)	UMNK88_RS26480	1	0	0	0
		faeD		UMNK88_RS26485	1	0	0	0
		faeE		UMNK88_RS26490	1	0	0	0
		faeF		UMNK88_RS26495	1	0	0	0
		faeG		UMNK88_RS26500	1	0	0	0
		faeH		UMNK88_RS26505	1	0	0	0
		faeI		UMNK88_RS26510	1	0	0	0
		faeJ		UMNK88_RS26515	1	0	0	0
	**Afimbrial adhesin AFA-I**	*afaD*	UPEC str. VR50 (NZ_CP011134.1)	ECVR50_RS16495	0	0	0	0
		afaC		ECVR50_RS16500	0	0	0	0
		afaB		ECVR50_RS16505	0	0	0	0
		afaA		ECVR50_RS16510	0	0	0	0
		afaE		ECVR50_RS16485	0	0	0	0
		draP		ECVR50_RS16490	0	0	0	0
	**Curli fimbriae**	*cgsG*	*Escherichia coli* str. K-12 substr. MG1655 (NC_000913.3)	b1037	1	1	1	1
		cgsF		b1038	1	1	1	1
		cgsE		b1039	1	1	1	1
		cgsD		b1040	1	1	1	1
		csgB		b1041	1	1	1	1

		csgA		b1042	1	1	1	1
		csgC		b1043	1	1	1	1
	***E. coli* laminin-binding fimbriae (ELF)**	*elfA*	EHEC str. 11368 (NC_013361.1)	ECO26_RS05610	1	1	1	0
		elfD		ECO26_RS05615	1	1	1	0
		elfC		ECO26_RS05620	1	1	1	0
		elfG		ECO26_RS05625	1	1	1	0
	**ECP**	*ecpR*	StxEAEC str. 2011C-3493 (NC_018658.1)	O3K_RS20165	1	1	1	1
		ecpA		O3K_RS20170	1	1	1	1
		ecpB		O3K_RS20175	1	1	1	1
		ecpC		O3K_RS20180	Point deletion	1	1	1
		ecpD		O3K_RS20185	1	1	Amber mutation (G to A)	1
		ecpE		O3K_RS20190	1	1	1	1
	**F1C fimbriae**	*focA*	UPEC str. CFT073 (NC_004431.1)	C_RS05815	0	0	0	1
		focI		C_RS05820	0	0	0	0
		focC		C_RS05825	0	0	0	1
		focD		C_RS05830	0	0	0	1
		focF		C_RS05835	0	0	0	1
		focG		C_RS05840	0	0	0	1
		focH		C_RS05845	0	0	0	1
	**Hemorrhagic *E. coli* pilus (HCP)**	*hcpA*	EHEC str. EDL933 (NC_002655.2)	Z_RS00570	1	1	1	1
		hcpB		Z_RS00565	1	1	1	1
		hcpC		Z_RS00560	1	1	1	1
	**P fimbriae**	*papX*	UPEC str. UTI89 (NC_007946.1)	UTI89_RS23655	0	0	0	0
		papG		UTI89_RS23660	0	0	1	0
		papF		UTI89_RS23665	0	0	1	0
		papE		UTI89_RS23670	0	0	1	0
		papK		UTI89_RS23675	0	0	1	0
		papJ		UTI89_RS23680	0	0	1	0
		papD		UTI89_RS23685	0	0	1	0
		papC		UTI89_RS23690	0	0	1	0
		papH		UTI89_RS23695	0	0	Point deletion	0
		papA		UTI89_RS23700	0	0	Inversion	0
		papB		UTI89_RS23705	0	0	1	0
		papI		UTI89_RS23715	0	0	1	0
	**S fimbriae**	*sfaC*		UM146_RS12845	0	0	0	0

		sfaB	AIEC str. UM146 (NC_017632.1)	UM146_RS12840	0	0	0	0
		sfaA		UM146_RS12835	0	0	0	0
		sfaD		UM146_RS12830	0	0	0	0
		sfaE		UM146_RS12825	0	0	0	0
		sfaF		UM146_RS12820	0	0	0	0
		sfaG		UM146_RS12815	0	0	0	0
		sfaS		UM146_RS12810	0	0	0	0
		sfaH		UM146_RS12805	0	0	0	0
		sfaY		UM146_RS12800	0	0	0	0
	**Type 1 fimbriae**	*fimB*	EHEC str. EDL933 (NC_002655.2)	Z_RS27690	1	1	1	1
		fimE		Z_RS27695	1	1	1	1
		fimA		Z_RS27705	1	1	1	1
		fimI		Z_RS27710	1	1	1	1
		fimC		Z_RS27715	1	1	1	1
		fimD		Z_RS27720	1	1	1	1
		fimF		Z_RS27725	1	1	1	1
		fimG		Z_RS27730	1	1	1	1
		fimH		Z_RS27735	1	1	1	1
**Adherence and Biofilm/Adherence factors**	**AatA, AIDA-I type**	*aatA*	APEC str. O1 plasmid pAPEC-O1-ColBM (NC_009837.1)	APECO1_RS26165	0	0	0	0
	**AIDA-I**	*aidA*	UPEC str. EC958 (NZ_HG941718.1)	EC958_RS21030	0	0	0	0
	**Antigen 43, AIDA-I type**	*agn43*	*Escherichia coli* str. K-12 substr. MG1655 (NC_000913.3)	b2000	1	0	1	1
	**Cah, AIDA-I type**	*cah*	EHEC str. EDL933 (NC_002655.2)	Z_RS05535	0	1	1	0
	**EaeH**	*eaeH/fdeC*	EHEC str. EDL933 (NC_002655.2)	Z_RS01730	1	1	Large deletion	1
	**Inverse autotransporter adhesin EaeX/Air**	*eaeX/air*	EAEC str. 042 (NC_017626.1)	EC042_RS21320	0	0	0	1
	**EhaA, AIDA-I type**	*ehaA*	EHEC str. EDL933 (NC_002655.2)	Z_RS01845	0	0	0	0
	**EhaB, AIDA-I type**	*ehaB*	EHEC str. EDL933 (NC_002655.2)	Z_RS02155	1	1	Amber mutation (C to A)	1
	**EtpA**	*etpA*	ETEC str. H10407 plasmid p948 (NC_017724.1)	ETEC_RS30895	0	0	0	0
	**Paa**	*paa*	EHEC str. 12009 (NC_013353)	ECO103_RS08940	1	0	0	0

	**AIDA-I type**	*tibA*	ETEC str. H10407 (NC_017633.1)	ETEC_RS11690	0	0	0	0
	**UpaG adhesin, trimeric AT**	*upaG/ehaG*	EHEC str. EDL933 (NC_002655.2)	Z_RS23640	1	1	0	1
	**UpaH, AIDA-I type**	*upaH*	UPEC str. 536 (NC_008253.1)	ECP_RS07185	1	0	1	1
**Invasion**	**Ibes**	*ibeA*	APEC str. O1 (NC_008563.1)	APECO1_RS24970	0	0	0	0
		ibeB/cusC		APECO1_RS02755	1	1	1	1
		ibeC		APECO1_RS22565	1	1	1	1
	**Tia/Hek**	*tia*	APEC str. O1 (NC_008563.1)	APECO1_RS16825	0	0	0	0
**Delivery system/T6SS and related effectors**	**T6SS-1**	*tssB*	EAEC str. 042 (NC_017626.1)	EC042_RS24170	0	0	0	0
		tssC		EC042_RS24175	0	0	0	0
		tssK		EC042_RS24180	0	0	0	0
		tssL		EC042_RS24185	0	0	0	0
		NA		EC042_RS24190	0	0	0	0
		hcp		EC042_RS24195	0	0	0	0
		tssH		EC042_RS24200	0	0	0	0
		NA		EC042_RS24205	0	0	0	0
		NA		EC042_RS28850	0	0	0	0
		vgrG		EC042_RS24215	0	0	0	0
		tle1		EC042_RS24220	0	0	0	0
		tli1		EC042_RS24225	0	0	0	0
		tli1		EC042_RS24230	0	0	0	0
		NA		EC042_RS24235	0	0	0	0
		NA		EC042_RS24240	0	0	0	0
		tssM		EC042_RS24245	0	0	0	0
		tssA		EC042_RS24250	0	0	0	0
		NA		EC042_RS24255	0	0	0	0
		tssF		EC042_RS24260	0	0	0	0
		tssG		EC042_RS24265	0	0	0	0
		tssJ		EC042_RS24270	0	0	0	0
		tssE		EC042_RS24275	0	0	0	0
		NA		EC042_RS24280	0	0	0	0
		NA		EC042_RS24285	0	0	0	0
		NA		EC042_RS24290	0	0	0	0
		NA		EC042_RS24300	0	0	0	0
		NA		EC042_RS24305	0	0	0	0
	**T6SS-2**	*hcp*		Z_RS01140	1	1	1	1

		NA	EHEC str. EDL933 (NC_002655.2)	Z_RS01145	1	1	1	1
		tssM		Z_RS01150	Amber mutation (C to T)	1	Large deletion	1
		tssA		Z_RS01155	1	1	0	1
		tagO		Z_RS01165	1	Large deletion	0	1
		tssH		Z_RS01170	1	Large deletion	0	1
		icmH/tssL		Z_RS01175	1	1	0	1
		tssK		Z_RS01180	1	1	0	1
		tssJ		Z_RS01185	1	1	0	1
		tagH		Z_RS01190	1	1	0	1
		tssG		Z_RS01195	1	1	0	1
		tssF		Z_RS01200	1	1	0	1
		tssE		Z_RS01205	1	1	0	1
		tssC		Z_RS01210	1	1	0	1
		NA		Z_RS01215	1	1	0	1
		tssB		Z_RS01220	1	1	0	0
		hcp		Z_RS01230	1	1	0	1
		vgrG		Z_RS01240	1	1	0	1
	**T6SS-3**	*tssB*	StxEAEC str. 2011C-3493 (NC_018658.1)	O3K_RS04340	1	0	0	0
		tssC		O3K_RS04345	1	0	0	0
		aaiC		O3K_RS04350	1	0	0	0
		NA		O3K_RS04355	1	0	0	0
		tssF		O3K_RS04360	1	0	0	0
		tssG		O3K_RS04365	1	0	0	0
		vgrG		O3K_RS04370	1	0	0	0
		NA		O3K_RS04375	1	0	0	0
		NA		O3K_RS04380	1	0	0	0
		tssA		O3K_RS04385	1	0	0	0
		tssJ		O3K_RS04390	1	0	0	0
		tssK		O3K_RS04395	1	0	0	0
		NA		O3K_RS04400	1	0	0	0
		NA		O3K_RS04405	1	0	0	0
		tssH		O3K_RS04410	1	0	0	0
**Delivery system/T3SS and related effectors**	**T3SS**	*escG*	EHEC str. EDL933 (NC_002655.2)	Z_RS23990	0	0	0	0
		escF		Z_RS23995	0	0	0	0
		cesD2		Z_RS24000	0	0	0	0
		espB		Z_RS24005	0	0	0	0

		espD		Z_RS24010	0	0	0	0
		espA		Z_RS24015	0	0	0	0
		sepL		Z_RS24020	0	0	0	0
		escD		Z_RS24025	0	0	0	0
		cesT		Z_RS24035	0	0	0	0
		cesF		Z_RS24050	0	0	0	0
		sepQ/escQ		Z_RS24060	0	0	0	0
		escP		Z_RS24065	0	0	0	0
		escN		Z_RS24075	0	0	0	0
		escV		Z_RS24080	0	0	0	0
		cesL		Z_RS24085	0	0	0	0
		escI/sctI		Z_RS24095	0	0	0	0
		escJ		Z_RS24100	0	0	0	0
		sepD		Z_RS24105	0	0	0	0
		escC		Z_RS24110	0	0	0	0
		cesD		Z_RS24115	0	0	0	0
		glrA		Z_RS24120	0	0	0	0
		glrR		Z_RS24125	0	0	0	0
		etgA		Z_RS24130	0	0	0	0
		escU		Z_RS24135	0	0	0	0
		escT		Z_RS24140	0	0	0	0
		escS		Z_RS24145	0	0	0	0
		escR		Z_RS24150	0	0	0	0
		espL		Z_RS24155	0	0	0	0
		cesAB		Z_RS24165	0	0	0	0
		escE		Z_RS24170	0	0	0	0
		ler		Z_RS24175	0	0	0	0
	**T3SS secreted effectors**	*cif*	EPEC str. E2348/69 (NC_011601.1)	E2348C_RS03860	0	0	0	0
		espB	EHEC str. EDL933 (NC_002655.2)	Z_RS24005	0	0	0	0
		espF	EHEC str. EDL933 (NC_002655.2)	Z_RS24055	0	0	0	0
		espFu/tccP	EHEC str. EDL933 (NC_002655.2)	Z_RS14410	0	0	0	0
		espG	EHEC str. EDL933 (NC_002655.2)	Z_RS24180	0	0	0	0
		espG2	EPEC str. E2348/69 (NC_011601.1)	E2348C_RS15645	0	0	0	0

		espH	EHEC str. EDL933 (NC_002655.2)	Z_RS24055	0	0	0	0
		espJ	EHEC str. EDL933 (NC_002655.2)	Z_RS14405	0	0	0	0
		espK	EHEC str. EDL933 (NC_002655.2)	Z_RS08525	0	0	0	0
		espL1	EHEC str. EDL933 (NC_002655.2)	Z_RS12905	Point insertion	Point insertion	Point insertion	1
		espL2	EHEC str. EDL933 (NC_002655.2)	Z_RS20300	0	0	0	0
		espL4	EHEC str. EDL933 (NC_002655.2)	Z_RS26190	1	1	1	1
		espM1	EHEC str. EDL933 (NC_002655.2)	Z_RS11830	0	0	0	0
		espM2	EHEC str. EDL933 (NC_002655.2)	Z_RS18375	0	0	0	0
		espN	EHEC str. Sakai (NC_002695.2)	ECs_1561	0	0	0	0
		espO1-1	EHEC str. Sakai (NC_002695.2)	ECs_1567	0	0	0	0
		espO1-2	EHEC str. Sakai (NC_002695.2)	ECs_1821	0	0	0	0
		espR1	EHEC str. Sakai (NC_002695.2)	ECs_2073	0	0	0	1
		espR3	EHEC str. EDL933 (NC_002655.2)	Z_RS14195	0	0	0	1
		espR4	EHEC str. EDL933 (NC_002655.2)	Z_RS14210	0	0	0	0
		espW	EHEC str. EDL933 (NC_002655.2)	Z_RS18385	0	0	0	0
		espX1	EHEC str. EDL933 (NC_002655.2)	Z_RS00120	Point deletion	Point deletion	Point deletion	1
		espX2	EHEC str. EDL933 (NC_002655.2)	Z_RS04570	0	0	0	0
		espX5	EHEC str. EDL933 (NC_002655.2)	Z_RS26490	1	Point insertion	1	1
		espX6	EHEC str. EDL933 (NC_002655.2)	Z_RS27845	0	0	0	1
		espY1	EHEC str. EDL933 (NC_002655.2)	Z_RS00315	0	0	1	1
		espY2	EHEC str. EDL933 (NC_002655.2)	Z_RS00375	0	0	1	1
		espY3	EHEC str. EDL933 (NC_002655.2)	Z_RS02420	0	0	0	1
		espY4	EHEC str. EDL933 (NC_002655.2)	Z_RS24535	0	0	0	1
		espZ	EHEC str. EDL933 (NC_002655.2)	Z_RS24090	0	0	0	0

		map	EHEC str. EDL933 (NC_002655.2)	Z_RS24045	0	0	0	0
		nleA	EHEC str. EDL933 (NC_002655.2)	Z_RS11885	0	0	0	0
		nleB	EHEC str. EDL933 (NC_002655.2)	Z_RS20305	0	0	0	0
		nleB2	EHEC str. EDL933 (NC_002655.2)	Z_RS04410	0	0	0	0
		nleC	EHEC str. EDL933 (NC_002655.2)	Z_RS04415	0	0	0	0
		nleD	EHEC str. EDL933 (NC_002655.2)	Z_RS04430	0	0	0	0
		nleE	EHEC str. EDL933 (NC_002655.2)	Z_RS20310	0	0	0	0
		nleE-2	EPEC str. E2348/69 (NC_011601.1)	E2348C_RS05730	0	0	0	0
		nleF	EHEC str. EDL933 (NC_002655.2)	Z_RS11870	0	0	0	0
		nleG-1	EPEC str CB9615 (NC_013941.1)	G2583_RS01745	0	0	0	0
		nleG-2	EPEC str CB9615 (NC_013941.1)	G2583_RS01740	0	0	0	0
		nleG2-2	EHEC str. EDL933 (NC_002655.2)	Z_RS10950	0	0	0	0
		nleG2-3	EHEC str. EDL933 (NC_002655.2)	Z_RS10100	0	0	0	0
		nleG-3	EPEC str CB9615 (NC_013941.1)	G2583_RS16475	0	0	0	0
		nleG5-1	EHEC str. EDL933 (NC_002655.2)	Z_RS10940	0	0	0	0
		nleG5-2	EHEC str. EDL933 (NC_002655.2)	Z_RS10110	0	0	0	0
		nleG6-1	EHEC str. EDL933 (NC_002655.2)	Z_RS30050	0	0	0	0
		nleG6-2	EHEC str. EDL933 (NC_002655.2)	Z_RS29975	0	0	0	0
		nleG6-3	EHEC str. EDL933 (NC_002655.2)	Z_RS18390	0	0	0	0
		nleG7	EHEC str. EDL933 (NC_002655.2)	Z_RS32300	0	0	0	0
		nleG8-2	EHEC str. EDL933 (NC_002655.2)	Z_RS18380	0	0	0	0
		nleH1	EHEC str. EDL933 (NC_002655.2)	Z_RS04420	0	0	0	0
		nleH2	EHEC str. EDL933 (NC_002655.2)	Z_RS11875	0	0	0	0
		nleL	EHEC str. EDL933 (NC_002655.2)	Z_RS08485	0	0	0	0

		tccP2	EHEC str. 12009 (NC_013353)	ECO103_RS12465	0	0	0	0
		tir	EHEC str. EDL933 (NC_002655.2)	Z_RS24040	0	0	0	0
**Delivery related proteins**	**Contact-dependent inhibition CDI system**	*cdiA*	AIEC str. UM146 (NC_017632.1)	UM146_RS22745	1	1	1	1
		cdiB	AIEC str. UM146 (NC_017632.1)	UM146_RS22750	1	1	1	1
	**Efa-1/LifA**	*lifA/efa1*	EHEC str. 12009 (NC_013353)	ECO103_RS26085	0	0	0	0
	**EspC**	*espC*	EPEC str. E2348/69 (NC_011601.1)	E2348C_RS15640	0	0	0	0
	**EspI, SPATE**	*espI*	EHEC str. 12009 (NC_013353)	ECO103_RS20080	0	0	1	0
	**EspP**	*espP*	EHEC str. EDL933 (NC_002655.2)	Z_RS28340	0	0	0	1
	**Intimin**	*eae*	EHEC str. EDL933 (NC_002655.2)	Z_RS24030	0	0	0	0
	**Pet**	*pet*	EAEC str. 042 plasmid pAA (NC_017627.1)	EC042_RS26410	0	0	0	0
	**Pic**	*pic*	StxEAEC str. 2011C-3493 (NC_018658.1)	O3K_RS23325	2	0	0	0
	**Sat**	*sat*	NMEC str. CE10 (NC_017646.1)	CE10_RS17895	0	0	0	0
	**ToxB**	*toxB*	EHEC str. EDL933 (NC_002655.2)	Z_RS28710	0	0	0	0
	**Tsh**	*tsh*	APEC str. O1 plasmid pAPEC-O1-ColBM (NC_009837.1)	APECO1_RS26050	0	0	0	0
	**Vacuolating autotransporter Vat**	*vat*	APEC str O1 (NC_008563.1)	APECO1_RS01475	0	0	0	0
**Exotoxin**	**Entero-Hemolysin**	*hlyD*	EHEC str. EDL933 (NC_002655.2)	Z_RS28485	Point deletion	0	0	1
		hlyB	EHEC str. EDL933 (NC_002655.2)	Z_RS28480	1	0	0	1
		hlyA	EHEC str. EDL933 (NC_002655.2)	Z_RS28475	1	0	0	1
		hlyC	EHEC str. EDL933 (NC_002655.2)	Z_RS28470	1	0	0	1
	**CDT**	*cdtA*	APEC str O1 (NC_008563.1)	APECO1_RS07740	0	0	0	0
		cdtB	APEC str O1 (NC_008563.1)	APECO1_RS07745	0	0	0	0
		cdtC	APEC str O1 (NC_008563.1)	APECO1_RS07750	0	0	0	0
	**CNF-1**	*cnf1*	AIEC str. UM146 (NC_017632.1)	UM146_RS22630	0	0	0	0
	**Enterotoxin SenB/TieB**	*senB*	AIEC str. UM146 plasmid pUM146 (NC_017630.1)	UM146_RS25260	0	0	0	0

	**Heat-labile toxin (LT)**	*eltA*	ETEC str. UMNK88 (NC_017640.1)	UMNK88_RS31860	1	0	0	0
		eltB	ETEC str. UMNK88 (NC_017640.1)	UMNK88_RS27635	1	0	0	0
	**Hemolysin**	*hlyE*	EHEC str. EDL933 (NC_002655.2)	Z_RS09085	Large deletion	1	Large insertion (IS4)	1
	**Shiga toxins (Stxs)**	*stx1A*	EHEC str. EDL933 (NC_002655.2)	Z_RS15680	1	0	1	0
		stx1B	EHEC str. EDL933 (NC_002655.2)	Z_RS15675	1	0	1	0
		stx2A	EHEC str. EDL933 (NC_002655.2)	Z_RS06790	0	0	1	1
		stx2B	EHEC str. EDL933 (NC_002655.2)	Z_RS06795	0	0	1	1
**Iron acquisition**	**Aerobactin**	*iutA*	StxEAEC str. 2011C-3493 (NC_018658.1)	O3K_RS23250	0	0	0	0
		iucD	StxEAEC str. 2011C-3493 (NC_018658.1)	O3K_RS23255	0	0	0	0
		iucC	StxEAEC str. 2011C-3493 (NC_018658.1)	O3K_RS23260	0	0	0	0
		iucB	StxEAEC str. 2011C-3493 (NC_018658.1)	O3K_RS23265	0	0	0	0
		iucA	StxEAEC str. 2011C-3493 (NC_018658.1)	O3K_RS23270	0	0	0	0
	**Chu**	*chuS*	EHEC str. EDL933 (NC_002655.2)	Z_RS23065	0	0	0	1
		chuA	EHEC str. EDL933 (NC_002655.2)	Z_RS23070	0	0	0	1
		chuT	EHEC str. EDL933 (NC_002655.2)	Z_RS23080	0	0	0	1
		chuW	EHEC str. EDL933 (NC_002655.2)	Z_RS23085	0	0	0	1
		chuX	EHEC str. EDL933 (NC_002655.2)	Z_RS23090	0	0	0	1
		chuY	EHEC str. EDL933 (NC_002655.2)	Z_RS23095	0	0	0	1
		chuU	EHEC str. EDL933 (NC_002655.2)	Z_RS23100	0	0	0	1
	**Iron-regulated element**	*ireA*	APEC str O1 (NC_008563.1)	APECO1_RS17010	0	0	1	0
	**Iron/manganese transport**	*sitA*	APEC str O1 (NC_008563.1)	APECO1_RS06095	0	0	0	0
		sitB	APEC str O1 (NC_008563.1)	APECO1_RS06090	0	0	0	0
		sitC	APEC str O1 (NC_008563.1)	APECO1_RS06085	0	0	0	0
		sitD	APEC str O1 (NC_008563.1)	APECO1_RS06080	0	0	0	0
	**Salmochelin siderophore**	*iroN/fepA*	AIEC str. UM146 (NC_017632.1)	UM146_RS12790	1	1	1	1

		iroE	AIEC str. UM146 (NC_017632.1)	UM146_RS12785	0	0	0	0
		iroD	AIEC str. UM146 (NC_017632.1)	UM146_RS12780	0	0	0	0
		iroC	AIEC str. UM146 (NC_017632.1)	UM146_RS12775	0	0	0	0
		iroB	AIEC str. UM146 (NC_017632.1)	UM146_RS12770	0	0	0	0
	**Yersiniabactin siderophore**	*ybtS*	StxEAEC str. 2011C-3493 (NC_018658.1)	O3K_RS09750	1	0	0	0
		ybtX	StxEAEC str. 2011C-3493 (NC_018658.1)	O3K_RS09745	1	0	0	0
		ybtQ	StxEAEC str. 2011C-3493 (NC_018658.1)	O3K_RS09740	1	0	0	0
		ybtP	StxEAEC str. 2011C-3493 (NC_018658.1)	O3K_RS09735	1	0	0	0
		ybtA	StxEAEC str. 2011C-3493 (NC_018658.1)	O3K_RS09730	1	0	0	0
		irp2	StxEAEC str. 2011C-3493 (NC_018658.1)	O3K_RS09725	1	0	0	0
		irp1	StxEAEC str. 2011C-3493 (NC_018658.1)	O3K_RS09720	1	0	0	0
		ybtU	StxEAEC str. 2011C-3493 (NC_018658.1)	O3K_RS09715	1	0	0	0
		ybtT	StxEAEC str. 2011C-3493 (NC_018658.1)	O3K_RS09710	1	0	0	0
		ybtE	StxEAEC str. 2011C-3493 (NC_018658.1)	O3K_RS09705	1	0	0	0
		fyuA	StxEAEC str. 2011C-3493 (NC_018658.1)	O3K_RS09700	1	0	0	0
**Others**	**Dispersin**	*aap*	StxEAEC str. 2011C-3493 plasmid (NC_018666.1)	O3K_RS26115	0	0	0	0
		aatP	StxEAEC str. 2011C-3493 plasmid (NC_018666.1)	O3K_RS26520	0	0	0	0
		aatA	StxEAEC str. 2011C-3493 plasmid (NC_018666.1)	O3K_RS26515	0	0	0	0
		aatB	StxEAEC str. 2011C-3493 plasmid (NC_018666.1)	O3K_RS26510	0	0	0	0
		aatC	StxEAEC str. 2011C-3493 plasmid (NC_018666.1)	O3K_RS26505	0	0	0	0
		aatD	StxEAEC str. 2011C-3493 plasmid (NC_018666.1)	O3K_RS26500	0	0	0	0

1, present; 0, absent
